# Machine learning in subsurface physical properties and lithofacies prediction in a mining context

**DOI:** 10.1038/s41598-025-11953-4

**Published:** 2025-07-21

**Authors:** A. Balaguera, M. Torné, R. Carbonell, A. Martí, J. Vergés, M. J. Jurado, P. Sánchez-Pastor, A. Farci, D. Davoise, S. Rodríguez

**Affiliations:** 1 Geosciences Barcelona, GEO3BCN, CSIC, Lluís Solé i Sabarís, s/n, Barcelona, 08028 Spain; 2 Earth and Ocean Dynamics Department, Faculty of Earth Sciences, University of Barcelona, Martí i Franquès, s/n, Barcelona, 08028 Spain; 3 Atalaya Mining, c/La Dehesa s/n. 21660 Minas de Riotinto, Huelva, Spain

**Keywords:** Machine learning, Rock properties prediction, Lithofacies classification, Massive sulfide deposits, Exploration mining and Iberian pyrite belt, Geophysics, Geology, Scientific data

## Abstract

To address energy challenges linked to decarbonization, geosciences are focusing more on advancing mineral resource exploration and exploitation. This study uses the Iberian Pyrite Belt, one of the world’s largest metallogenic provinces, as a test site to: (a) develop predictive models of physical properties of rock (PPR) and (b) classify lithological units based on these PPR. Over 1,000 surface rock samples and six boreholes from the Riotinto mine were analyzed, providing a comprehensive dataset. A robust quality control process, aided by Machine Learning models (ML), refined the PPR data, ensuring accuracy and reliability. Both traditional statistical models and cuttingedge ML models were used for PPR prediction and lithofacies classification. Our study revealed that geological evolution can lead to significant overlaps in PPR across different lithologies, making traditional models insufficient for accurate predictions. However, ML models, such as Random Forest, XGBoost, k-Nearest Neighbors, and Support Vector Regression, demonstrated over 80% accuracy in predicting PPR and classifying lithofacies. This approach redefines how lithofacies are identified and establishes an innovative methodology for subsurface lithological characterization. Results highlight the potential of ML models in mining and geology, paving the way for more accurate 3D characterization of lithological units through integrating geophysical data and direct measurements.

**Supplementary Information:**

The online version contains supplementary material available at 10.1038/s41598-025-11953-4.

## Introduction

Machine learning (ML) techniques are revolutionizing mineral exploration and exploitation by addressing critical challenges of sustainability, efficiency, and resource estimation in geologically complex settings^1–7^. This transformation is particularly relevant as global demand for critical minerals, driven by the energy transition and technological advancements, continues to rise^8,9^^10,11^.,. Effective decision-making in mining, geophysics, and environmental management relies on accurate subsurface modeling, which requires robust characterization of physical rock properties such as density, seismic velocities, and porosity. However, traditional models often fail to account for the complex, nonlinear relationships inherent in heterogeneous geological settings, underscoring the possibilities of ML driven approaches capable of identifying hidden patterns.

In heterogeneous geological settings, relationships between surface observations and subsurface conditions are often obscured by tectonics and lithological variability, complicating resource estimation and exploitation strategies^12,13^. ML offers a solution by identifying hidden patterns and improving lithological classification, even in highly heterogeneous media^14^.

Recent advancements in computational sciences and ML techniques, including both deep learning (DL) and shallow geophysical characterization, have introduced powerful tools for improving lithological classification through the integration and interpretation of PPR data. These new developments are capable of extracting information from large volumes of data and identify hidden relationships^5,15^.When trained with representative datasets, ML approaches provide objective, reproducible and scalable schemes, that often outperform traditional interpretive methods in terms of consistency and flexibility^16,17^.

While DL architectures have shown remarkable success in fields such as image recognition, natural language processing, and time-series forecasting, their strengths lie in handling unstructured or high-dimensional data types (e.g., images, audio, sequential logs).These models typically require large, diverse, and densely sampled datasets to perform effectively and avoid overfitting^1–4,16,17^. In contrast, the dataset used in this study comprises structured, tabular physical property measurements—specifically density, porosity and P-wave velocity—obtained from approximately 1000 rock samples and 6 borehole intervals. These variables are continuous, low-dimensional, and domain-specific, which makes them well suited for more conventional ML algorithms (e.g., Random Forests, XGBoost) which are known for their interpretability and robustness, especially when working with small datasets. In this context, DL models not only offer limited advantages but also carry a higher risk of overfitting and reduced transparency. Furthermore, in subsurface characterization for mining applications, data acquisition is often constrained by cost, access, and geological heterogeneity, making traditional ML approaches offer a practical and reliable alternative for predictive modeling and lithological classification.

In the of mineral exploration continuous well logs commonly used in the oil and gas industry are generally absent, therefor, the supervised integration of surface and borehole datasets constitutes a significant methodological innovation. Previous studies have seldom merged surface and subsurface data within a single predictive framework; instead, they have typically relied on independent approaches such as geological mapping, geophysical surveys, geochemical sampling, or dense drilling campaigns to infer subsurface conditions (Table S1). In contrast, our study applies ML to combine petrophysical measurements from surface samples with borehole records, enable the extrapolation of empirical rock-property relationships to depth and cross-validating these predictions against borehole data. By leveraging an unusually comprehensive dataset (~ 1000 surface samples and six boreholes), this work illustrates how surface-derived information can effectively inform subsurface modeling, even in the absence of continuous downhole logs.

Equally notable is the capacity of this integrated ML approach to achieve realistic lithofacies classification in a setting characterized by sparse and spatially discontinuous data. Traditional linear or univariate classification methods often fail in such heterogeneous environments, where physical property ranges of different rock types overlap considerably. By capturing non-linear, multivariate patterns, the supervised models generate geologically coherent facies predictions that match borehole observations, offering a level of accuracy and spatial resolution that previous approaches could not attain under similar constraints. This demonstrates that, even with limited input data, ML enables robust and interpretable subsurface predictions, offering a practical advancement over earlier mining applications that did not exploit the integration of surface and borehole information.

Importantly, this work favors the acquisition of continuous log and imaging data within the mining industry. Such developments would enable the application of more advanced ML techniques, including DL, once the appropriate data conditions, such as a larger amount of rock samples, richer feature spaces, or image-based inputs are met. In this way, this study not only addresses immediate and practical challenges using appropriate methodology but also contributes, meaningfully, to bridge the gap between AI methodologies and their practical implementation in the this sector.

**Fig. 1:**
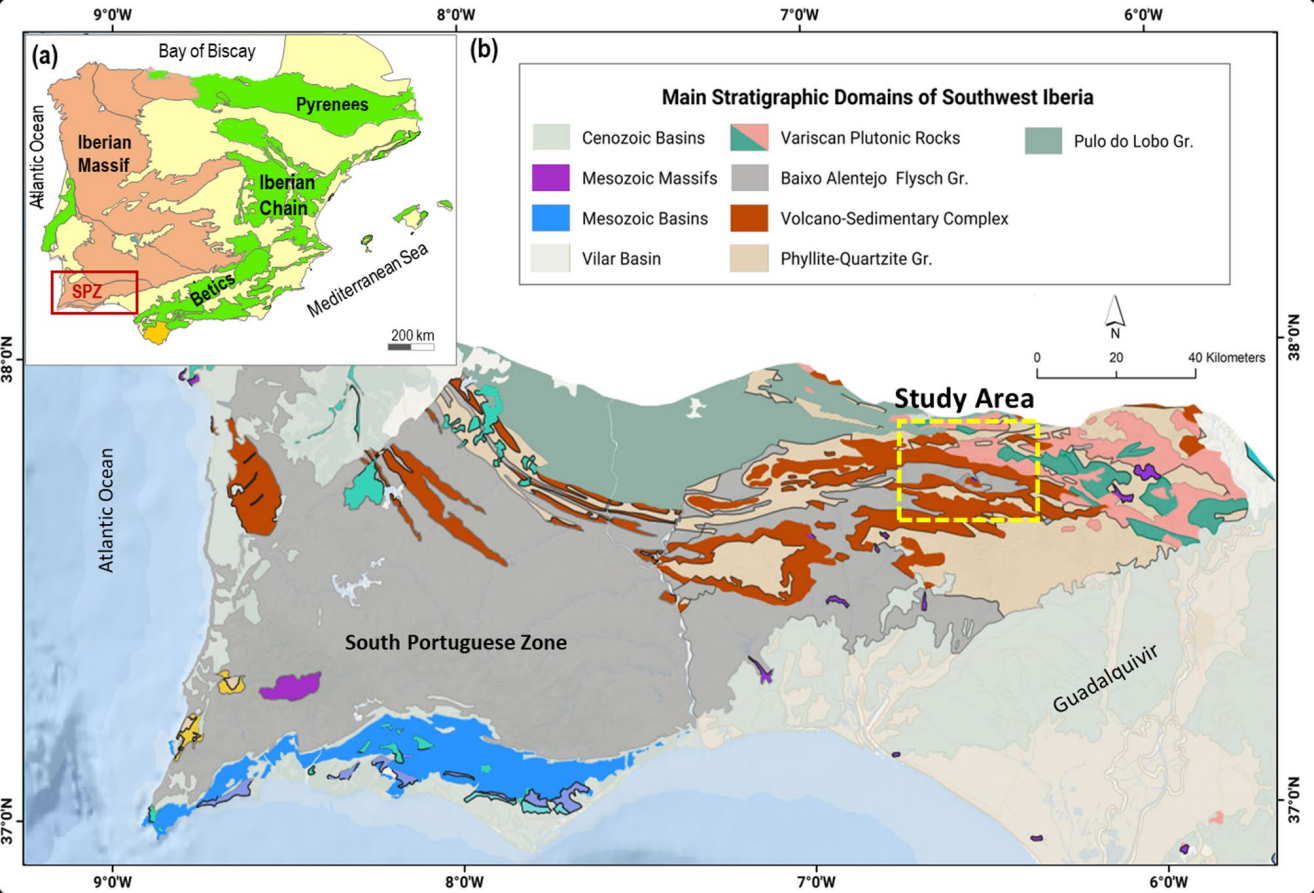
Location map. **a** Simplified tectonic map of Iberia highlighting the South Portuguese Zone (SPZ), within a red box. Generated using GMT 6.0.023, https://www.generic-mapping-tools.org/**b** Geological map of the South Portuguese Zone with emphasis on the Iberian Pyrite Belt. The yellow box indicates location of the study area. Geological data taken from open license24 and generated using QGIS Version 3.16.14, https://www.qgis.org/.

The study area is located within the Iberian Pyrite Belt (IPB) in the southwest of the Iberian Peninsula (Fig. 1). This region, stretching approximately 240 km in length and 35 km in width, forms an arc and is part of the South Portuguese Zone (SPZ), one of the best-exposed fragments of the Variscan orogeny^18^. The IPB is one of the most important volcanogenic massive sulfide (VMS) districts globally, with reserves exceeding 1600 Mt and approximately 2000 Mt of low-grade stockwork, having been mined for 5000 years^19–22^. Its long exploitation history underscores its economic and strategic importance, yet significant gaps remain in the geological understanding of the region, particularly in areas beyond those that have been actively mined.

Recognizing the global significance of the IPB and the importance of exploring beyond the exploited areas, this study aims to analyze PPR in complex geological settings from a petrophysical perspective.To achieve this, we propose an innovative methodology capable of predicting rock types from surface rock samples and applying these relationships to subsurface data, offering a significant advancement in geological and mining studies. By bridging the gap between surface and subsurface data, the proposed approach enhances our understanding of the geological characteristics of the Iberian Pyrite Belt (IPB) and has far-reaching implications for exploration strategies in mineral-rich regions. This methodology not only advances our knowledge of the geomechanical behavior of rocks but also helps the assessment of open-pit mines and tailings stability, addressing critical aspects of operational safety and environmental stewardship. Furthermore, its broad applicability extends beyond the IPB, providing valuable insights into mineral deposit assessment and geological understanding on a global scale. By enabling more effective exploration in under-explored areas, it improves the prediction of rock types from surface samples and translates these insights into more informed subsurface geological models. Ultimately, this approach benefits the geological and mining industries by optimizing exploration and exploitation strategies while also bolstering geotechnical risk management and decision-making in mining projects worldwide.

### Geological context

**Fig. 2:**
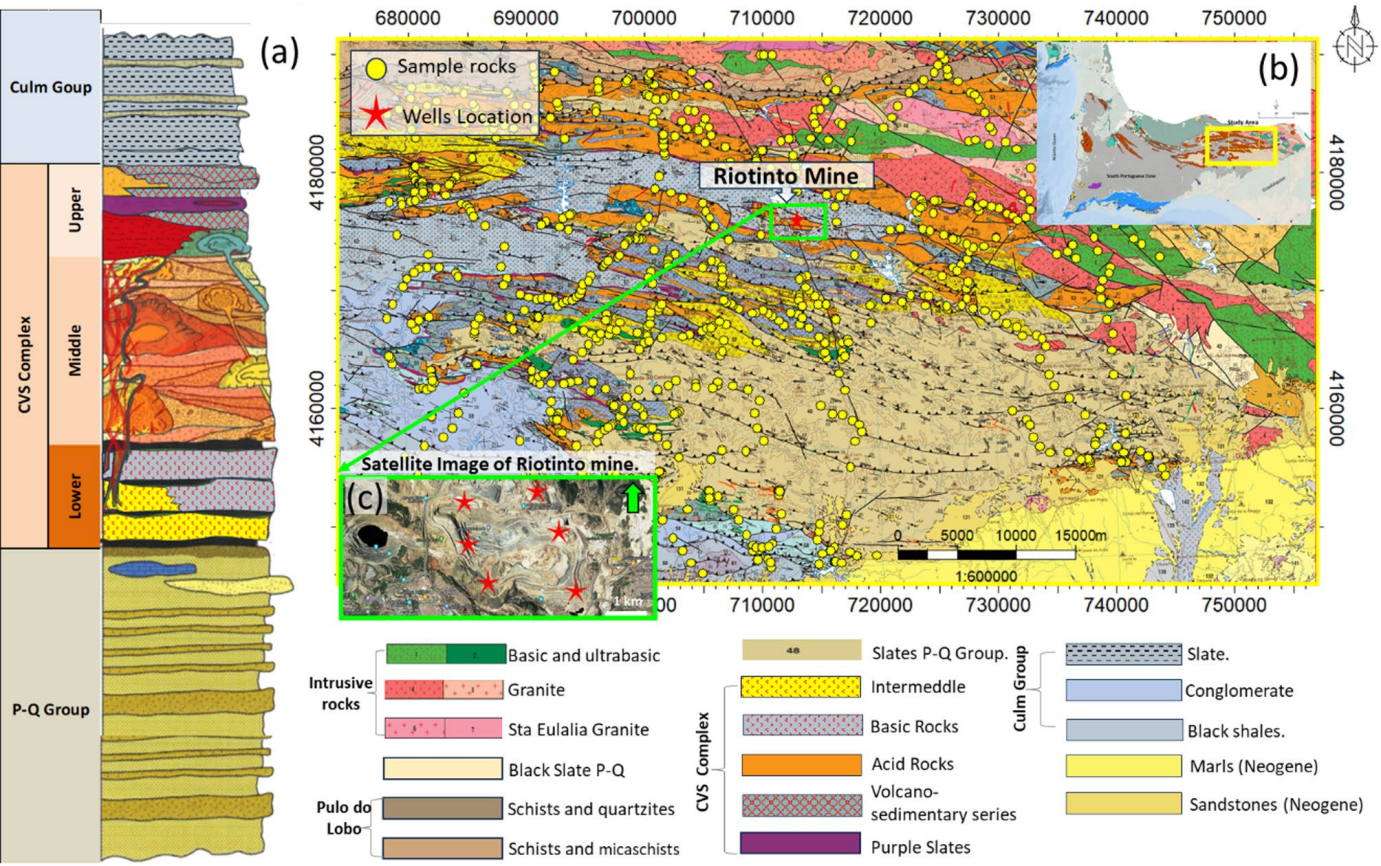
Geological information. **a** Schematic stratigraphic column of the study area. (Adapted from^25^). **b** Geological map corresponding to the study area.Geological data taken from open license^26^ and generated using QGIS Version 3.16.14, https://www.qgis.org/26 showing the location of rock samples (yellow circles) and (c) satellite image of the Riotinto Mine indicating borehole location (red stars) within the bright green box (bottom center)

The Riotinto Mine demonstration site is situated in the southern region of the Variscan Iberian Massif, within the Iberian Pyrite Belt (IPB). This massif constitutes a significant portion of the former Iberian Platet (Fig. 1). The IPB is one of the world’s largest concentrations of VMS deposits. It exhibits an arch shape geometry approximately 240 km long and 35 km wide between (Figs. 1b and 2b). The belt is predominantly composed of volcanic and sedimentary rocks dating from the Late Devonian to Pennsylvanian, and hosts over 80 VMS deposits^19,20^.

Geologically, the IPB is characterized by three main lithostratigraphic units: the Phyllite-Quartzite Group (P-Q Group), the Volcanic-Sedimentary Complex (CVS), and the Culm or Baixo Alentejo Flysch Group^27,28^(Fig. 2a).

- **Phyllite-Quartzite Group (P-Q Group)**: The oldest formation, predominantly exposed in the central-southern area (Fig. 2b), comprises metarenites and slates, with thicknesses reaching up to 2,000 meters^29^. Quartzites and quartzarenites within this group exhibit variable porosities (φ), while fossiliferous slates contain sericite, quartz, and chlorite. These deposits suggest a shallow continental shelf environment evolving towards higher-energy facies, such as sand bars and debris flows^30^.
- **Volcanic-Sedimentary Complex (CVS)**: Overlying the P-Q Group, the CVS is a heterogeneous sequence characterized by abrupt facies changes, with thickness varying significantly from 100 to 1,300 meters^19^ (Fig. 2a). Its lithological composition includes volcanic and sedimentary rocks such as shales, cherts, exhalites, and massive sulfides^31^. This sequence is attributed to crustal extension and partial melting of continental crust, subdivided into three units: Lower CVS, consisting of submarine basalt flows and black shales; Middle CVS, dominated by rhyolites and dacites; and Upper CVS, composed of acidic volcanic rocks and radiolarites, indicative of an anoxic outer platform environment20.
- **Culm Group (Baixo Alentejo Flysch Group)**: A detrital sequence marking the end of Carboniferous sedimentation (~ 298.9 Ma), it contains sedimentary rocks derived from reworking of older formations^32^.This group reflects basin deepening and is associated with SW-verging folds and thrusts, with an estimated thickness exceeding 1,000 m.

The area also exhibits a complex tectonic and magmatic evolution, with several intrusive units (Fig. 2a). These include Basic and Ultrabasic Units (Late Devonian to Early Carboniferous) associated with a magmatic arc and compressive tectonics, as well as post-orogenic granites (Middle to Late Carboniferous) emplaced under extensional conditions^20,27^. These units highlight the region’s diverse geological history and role as a significant mineral resource hub.

### Data and methods

The database used in this contribution consists of values of physical properties of rocks obtained from laboratory measurements of 1050 outcrop rock samples (Fig. 2b) as well as from subsurface measurements derived from boreholes (Fig. 2c). This dual-source dataset represents an unprecedented resource in the conventional mining operations (exploration, exploitation, etc.).In them petrophysical data are not considered, and if acquired they are usually fragmented, or limited to narrow spatial domains. Compiling such a comprehensive and spatially distributed petrophysical dataset has required significant effort across multiple field campaigns and previous studies. What distinguishes this dataset is not only its volume, but also its integration of both surface and subsurface data, enabling a more holistic characterization of the mineral system under investigation. Surface-derived measurements, collected through systematic field sampling and subsequent laboratory testing, were instrumental in capturing spatial variability in physical properties across the different lithological units. The borehole data provided continuous depth profiles and served as a baseline reference for model calibration and validation, allowing for the assessment of ML model performance under real-world subsurface conditions.

The combination of these two data types, in this contributionleverages a structurally diverse, high-quality dataset that supports robust feature-target relationships—essential for training and validating predictive models. This methodological approach provides an important step forward in developing scalable workflows for lithological classification in mineral exploration, particularly in settings where access to continuous logging or imaging technologies remains limited.

**Fig. 3:**
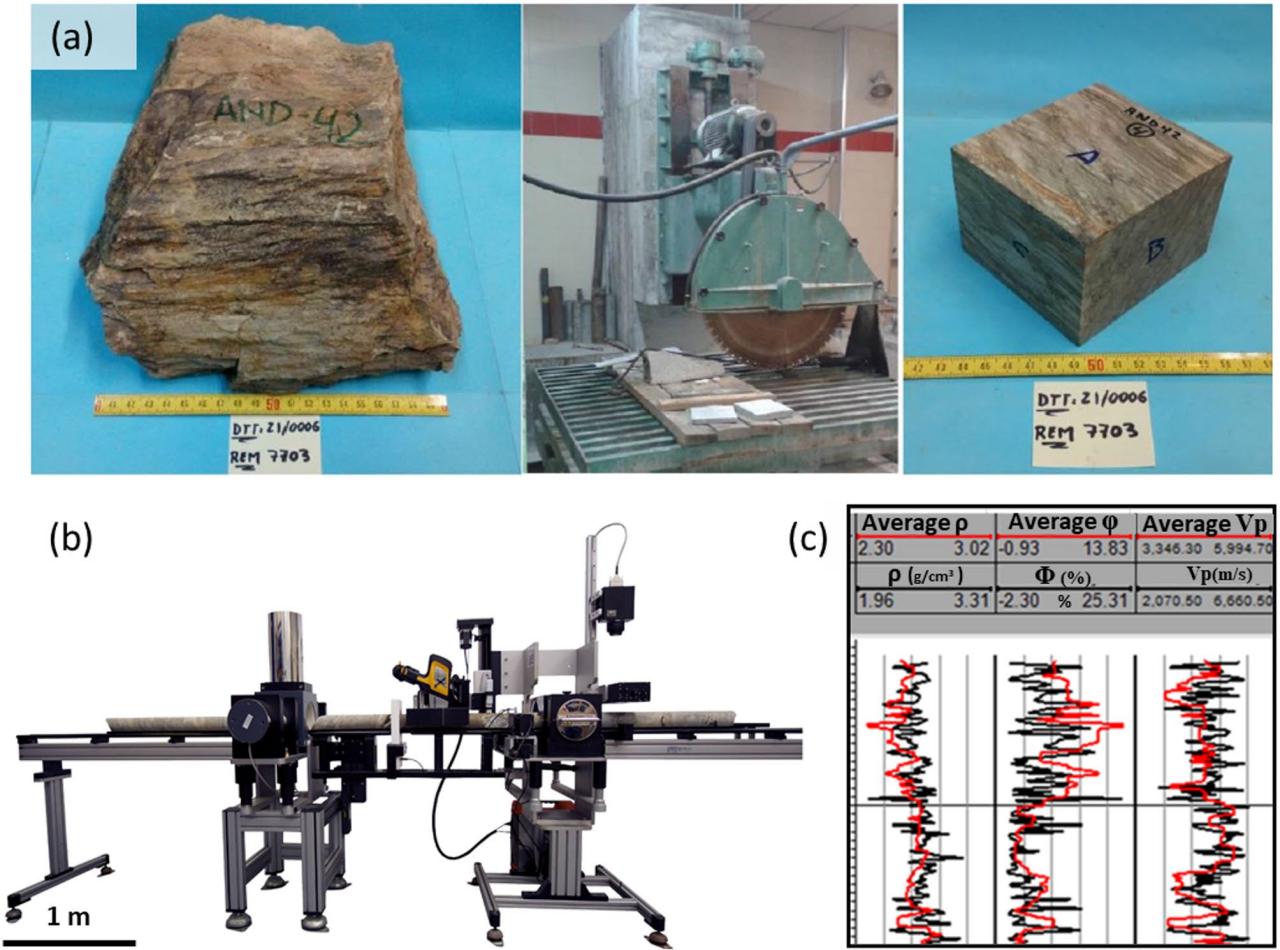
Rock samples and measurements. **a** Preparation of standard rock samples. **b** Measurement instrument for PPR (GeoTek),. (Adapted from^33^). y **c** Standard values of PPR ρ (g/cm³), φ (%), and Vp (m/s) for the first 60 samples

Surface rock samples were collected by the Geological and Mining Institute of Spain (IGME-CSIC)(Fig. 3a). The physical properties were measured using a Multi-Sensor Core Logger (MSCL-S) instrument from GeoTek (Fig. 3b). The resulting database includes the lithological description of the rock samples, its total porosity -φ- (%), P-wave velocity -Vp- (m/s), apparent density -ρ- (g/cm^3^), (Fig. 3c). Subsurface data included six exploratio boreholes, each reaching depths close to 1000 m (true vertical depths, TVD) within the Riotinto mine. These boreholes provided apparent density measurements and detailed lithological descriptions.

This research proposes aims touse e surface PPR data to predict petrophysical properties of the subsurface, enabling the classification of rock types in areas where a single petrophysical property is available. The latter estimated through direct or indirect methods. The approach complements conventional methods, such as geological and chemical mapping^34,35^geophysical studies^36^remote sensing^37^and dense borehole drilling grids.

. Leveraging surface data and ML models, through the proposed process a data informed guess of key subsurface properties such as ρ, φ, and Vp is carried out. This approach aligns with the growing demand for sustainable mining practices, necessary for meeting the increasing demand for strategic minerals like copper and lithium^38–40^. The methodology reveals how surface-derived data can be effectively extrapolated to depth, offering a viable solution for exploration in complex geological settings.

**Fig. 4:**
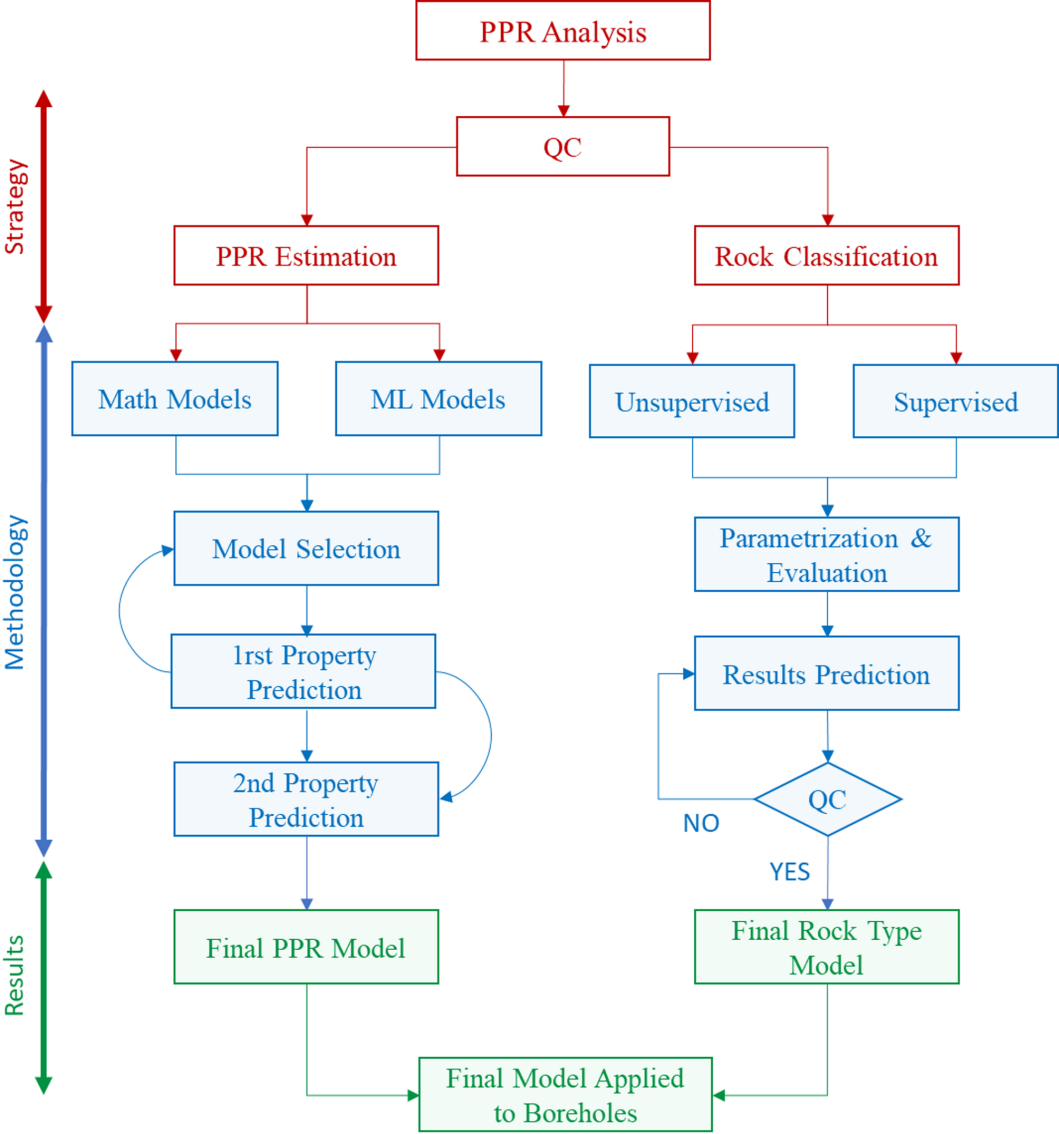
Workflow used in this study divided into three distinct blocks: Strategy, Methodology, and Results (red blue and green respectively)

The proposed methodology employs ML schemes to develop PPR models, through a three-stage workflow (Fig. 4). Given the structured nature and size of the dataset, shallow ML techniques were preferred. DL architectures generally require significantly larger and more complex datasets to generalize effectively and avoid overfitting^4,16,17^. First, the surface database is curated. Second, mathematical models are applied to evaluate and compare traditional approaches with ML based methods for predicting petrophysical properties and classifying lithofacies within geological units. Finally, the methodology is tested and validated using borehole data from the Riotinto mine to evaluate its consistency and accuracy.

The database of physical property values for the different rocks consists of a table with: the sample number, its physical properties, its geographical location, lithological characterization, lithofacies description. The location provides essential information about the stratigraphic unit or formation to which the sample belongs, determined by referencing its location on a geologic map. The QC analysis of the physical property values (ρ, φ and Vp) revealed the existence of singular values. Values that fell beyond a 90% confidence interval were identified and considered outliers. New values for these outliers were then estimated using linear regression models and, simple multi-layer perceptron neural network models (e.g., two layers with four neurons). The improvement was quantified by calculating the root mean squared error (RMSE) and, coefficient of determination (R²) values of the predicted properties.

### Mathematical models/Functional dependencies

The relationships between ρ, Vp and φ were analyzed for each geological unit to identify functional dependencies (linear or non-linear) dependencies. Once the independent variable with the highest correlation with ρ was identified, a second-order prediction was advanced, in which mathematical relationships were further explored to estimate φ or Vp. The analysis aimed to identify the mathematical relationship with the lowest error and highest predictive accuracy. These included logarithmic (Log), quadratic (Squared), inverse (Inv), square root (Sqrt), and trigonometric functions.

This analysis is grounded in previous geological studies that highlight the necessity of exploring non-linear relationships. For instance, Gardner^41^ in 1974 proposed empirical relationships between density and Vp, providing a functional link between physical properties. Subsequent research, such^42^ refined these relationships by accounting for slate content and fluid saturation effects, demonstrating the significant influence of geological factors on such correlations. Moreover, Wyllie^43^ in 1958 introduced the time-average equation, establishing a basis for approximating porosity using acoustic velocities. More recently, Carrasquilla^44^ in 2022 emphasized the importance of applying quadratic and logarithmic transformations to enhance the prediction of new PPR values in subsurface units characterized by high heterogeneity. These studies reveal the robust correlations (relationshios) between these properties and their dependence on the specific geological units.

Building on the theoretical exploration of potential linear or non-linear relationships, the functional relationship with the highest metrics was determined. Statistical metrics such as PCC, MSE, MAE, and R² were used to evaluate the robustness of these relationships (Table S2, supplementary material) and compare them with results from more advanced ML models.

### ML models

When functional relationships between φ, Vp, and ρ were unclear or metrics were low, supervised and unsupervised ML models were applied. In particular, for PPR estimation, data normalization using Z-score transformations^45,46^ facilitated the analysis of attribute behavior through multi-plot graphs. To ensure the predictive models focus on the most significant relationships, the target variable was selected as the property with the highest correlation coefficient with other attributes (Vp and φ), often ρ. This decision was based on Pearson correlation (PCC) analysis, as well as feature importance assessments derived from hierarchical models and permutation-based methods, and most consistent influence across models was used as the primary predictor. The Z-score normalization was applied to ensure that all features contributed equally to the model training process, minimizing biases caused by varying data scales^46^.

Data from each stratigraphic unit (P-Q Group, Lower, Middle, and Upper CVS, and Culm Group) were divided into training (75%), validation (20%), and test (5%) sets, maintaining a proportional representation of lithologies. A stratified sampling strategy was employed to ensure that each lithological class was represented in all subsets, as this approach preserves the diversity of the dataset and mitigates the risk of sampling bias^45^.The ML models applied included Random Forest Regression (RFR), Support Vector Regression (SVR), k-Nearest Neighbors (KNN), and Extreme Gradient Boosting (XGBoost).

Each ML model was optimized by tuning specific hyperparameters according to the algorithm used (Table 1), employing GridSearchCV^47^ for this purpose. GridSearchCV systematically evaluated all possible combinations of hyperparameter values specified in a parameter grid, ensuring the identification of the optimal configuration. For instance, in the RFR for estimating φ from ρ in the Culm geological unit (Table 1), parameters such as the number of estimators, maximum tree depth, and minimum samples per split were iteratively tested to enhance model performance.

For each geological unit, a detailed evaluation of ML model performance was conducted by testing a range of hyperparameter values, using ρ as the independent variable and Vp and φ as dependent properties. For validation, the Leave-One-Out Cross-Validation (LOOCV) method was used, particularly suitable for small datasets due to its exhaustive evaluation of model performance^45^. Importantly, the 20% validation set, independent from training, was used in combination with LOOCV and GridSearchCV during model tuning, providing robust generalization metrics and helping prevent overfitting.

This process systematically left out individual data points as validation sets while iteratively training on the remaining data. LOOCV provided robust generalization metrics and ensured reliable performance evaluation for each model. This iterative process was facilitated by the Scikit-learn Python library^48^. As a final quality control (QC) step regarding the performance of the different ML models, a 5% test set was kept hidden and used to verify the accuracy of the models. Although optional, the blind test reinforces the robustness of the methodology. Like the other subsets, it followed a stratified sampling scheme to preserve lithological representativeness. This final test phase, while not essential, acts as a complementary quality control step that strengthens the robustness of the validation scheme to ensure that the model’s performance is not a result of overfitting and that it generalizes well to entirely new data.

Statistical metrics such as Mean Squared Error (MSE), Mean Absolute Error (MAE), and Coefficient of Determination (R²) were applied to assess model accuracy, with results summarized in Table S3. R² was particularly valuable for quantifying the proportion of variance explained by the model, while MSE and MAE provided insights into prediction error magnitude. The process was repeated first to predict one property per geological unit, and subsequently to predict a third property, using the two previous properties as predictors. This approach help identify the best performing models for each property^49–51^A summary of results is provided in Table 1 and S2.

**Table 1:** Range of hyperparameters for the best-performing supervised ML models used in PPR prediction for each main geological unit.

		Objective	Attribute Variable	Best ML Model	Hyperparameters	Range of Values
		Variable				
Culm Group		φ	ρ	RFR	Minimum samples split	[2, 4, 7, 10]
					Minimum samples leaf	[1, 2, 5, 10, 20]
					Maximum tree depth	[5, 10, 15, 20]
		Vp	φ, ρ	SVR	Kernel	[Sigmoidal, Radial, Polynomial, Linear]
					Regularization	[1, 5, 10, 15, 20]
					Gamma	[0.001, 0.0125, 0.05, 0.5, 1]
CVS Complex	Upper	φ	ρ	XGBoost	n_estimators	[25, 50, 100, 150]
					Learning rate	[0.01, 0.1, 0.5]
					Maximum child weight	[5,8, 9, 10, 15]
					Min_samples_leaf	[0.5,1, 2, 4]
					Maximum tree depth	[2, 3, 4, 5
		Vp	φ, ρ	RFR	Minimum samples split	[2, 3, 5, 7]
					Minimum samples leaf	[1, 2, 5, 10, 20]
					Maximum tree depth	[5, 10, 15, 20]
	Middle	φ	ρ	KNN	N_neighbors	[3, 5, 6, 7, 8, 9]
					weights	[‘uniform’,‘distance’]
					algorithm	[‘auto’,‘ball_tree’,‘kd_tree’,‘brute’]
					leaf_size	[20,30, 40, 50]
					distance type	[Manhattan, Euclidiana]
		Vp	φ, ρ	XGBoost	n_estimators	[15, 25, 50, 100]
					Learning rate	[0.05, 0.1, 0.5,1]
					Maximum child weight	[2, 5,8, 9, 10, 15]
					Min_samples_leaf	[1, 2, 4,5]
					Maximum tree depth	[0.5,1, 2, 3, 4]
	Lower	Vp	ρ	RFR	Minimum samples split	[2, 4, 7, 10]
					Minimum samples leaf	[1, 2, 5, 10, 20]
					Maximum tree depth	[5, 10, 15, 20]
		φ	Vp, ρ	RFR	Minimum samples split	[2, 4, 7, 10]
					Minimum samples leaf	[1, 2, 5, 10, 20]
					Maximum tree depth	[5, 10, 15, 20]
PQ Group		φ	ρ	RFR	Minimum samples split	[2, 3, 5, 7]
					Minimum samples leaf	[1, 2, 5, 10, 20]
					Maximum tree depth	[5, 10, 15, 20]
		Vp	φ, ρ	SVR	Kernel	[Sigmoidal, Radial, Polynomial, Linear]
					Regularization	[1, 5, 10, 15, 20]
					Gamma	[0.001, 0.0125, 0.05, 0.5, 1]

### Rock type classification

Assigning labels to data items is one of the processing tasks for which ML models are used^15–17^. In this study, classification models were developed using the original dataset to ensure the highest accuracy and reliability, consistent with the goal of minimizing uncertainties in subsurface property predictions. As part of the quality control process, these models were compared with results obtained using PPR-predicted values, showing similar outcomes. The original dataset was prioritized to avoid propagating uncertainties associated with PPR prediction. This analysis employed both supervised ML models -Random Forest Classification (RFC), Classification and Regression Trees (CART), Decision Tree Classifier (ID3), and K-Nearest Neighbors (KNN)- and unsupervised ML models, including K-Means Clustering (K-Means) and the Gaussian Mixture Model (GMM).

A detailed summary of the hyperparameters and their respective ranges of values evaluated for each model is provided in Table S3 of the supplementary material. For supervised methods, hyperparameters were systematically optimized using GridSearch and Leave-one-out cross-validation (LOOCV) was implemented as the primary validation approach because it maximizes data utilization and provides robust model evaluation. Additionally, data partitions (75% training, 20% validation, and 5% test) were created using a stratified sampling approach, ensuring a minimum representation of 20% for each lithological class across all subsets. This methodology minimized sampling biases, preserved the lithological variability, and ensured robust and consistent evaluation of the models. As in the regression models, the 5% test subset was kept entirely independent and served as a complementary blind test.

For unsupervised methods, parameters such as n_components, covariance_type, and init_params were analyzed for GMM, with the optimal number of components determined using Akaike Information Criteria (AIC) and Bayesian Information Criteria (BIC). For K-Means, parameters such as n_clusters and init were evaluated, with the number of clusters optimized using inertia and silhouette analysis (Table S4 and Fig S4). The objective of this phase was to evaluate the feasibility of using PPRs as reliable tools for rock classification, thereby reducing uncertainty, particularly in the IPB. Documented lithologies were initially classified into three primary lithofacies: Slate-Volcaniclastic & Polygenetic Metasedimentary, Acidic & Intermediate Igneous Rocks, and Basic Igneous Rocks. Subsequently, two of these primary lithofacies (Slate-Volcaniclastic & Polygenetic Metasedimentary, and Acidic & Intermediate Igneous Rocks) were further subdivided, resulting in a total of five main lithofacies. This hierarchical classification approach follows strategies that prioritize identifying broad trends before refining into detailed subsets, as proposed in recent studies on lithofacies classification^52^. Building upon this approach, with the identification of the best classification models for the five lithofacies, additional QC was performed by iterating until statistical stability was reached. This step aimed to further evaluate the robustness and stability of the classification metrics across varied initial conditions, ensuring the reliability and reproducibility of the results. The classification aimed to maximize detail while maintaining an uncertainty margin below 30%.

### Prediction of PPR and lithofacies: the Riotinto case study

In the final phase, the developed PPR prediction and lithofacies classification models were tested in two distinct scenarios using borehole data from the Riotinto mine. The first scenario focused on exploration boreholes, to predict porosity, P-wave velocity, and lithofacies at depth based on apparent density samples. The second scenario involved a geotechnical borehole with a continuous Vp log, where the models were employed to estimate density and porosity. These estimates were subsequently validated against laboratory measurements.

Before applying the ML models, empirical transfer functions were developed to bridge differences between surface and subsurface domains, as the models were trained on surface data. Apparent ρ values from boreholes were analyzed by geological unit as a function of depth to identify gradients in property distributions. For units with clear mathematical relationships between PPRs and depth, these functions were directly applied. In units lacking such relationships, adjustments were made by correcting the drift in average property distributions.

## Results

### PPR and lithology distribution

Our analysis classified 22 primary lithologies across the P-Q Group, CVS Complex, and Culm Group into five main lithofacies, based on geological characteristics and compositional similarities. For instance, lithologies such as basalt, gabbro, diabase, and diorite were grouped into the Basic Igneous Rocks lithofacies due to their shared geochemical and mineralogical compositions. The identified lithofacies include Slate-Volcaniclastic, Polygenetic Metasedimentary, Acidic Igneous Rocks, Intermediate Igneous Rocks, and Basic Igneous Rocks. The dominant lithologies within each unit confirm the accuracy and representativeness of the lithofacies classification (Fig. S1a). For example, the Culm Group and Upper CVS are predominantly composed of Slate-Volcaniclastic lithofacies, primarily consisting of slate, tuff, and tuffite^28^. In contrast, the Middle CVS is characterized by acidic and intermediate igneous lithofacies (rhyolites and dacites), while the Lower CVS comprises the Basic Igneous lithofacies (basalts)^53^. The P-Q Group predominantly includes slates and sandstones, aligning with the Slate-Volcaniclastic lithofacies^54^. These distributions validate the classification framework and its alignment with the study area’s geological context (Fig. S1a and S1b in the suplementary materia).

Of the 1,050 analyzed rock samples, 32 outliers in PPR values were identified within a 90% confidence interval. These anomalies, originating from various lithologies, were recalculated as described in the data and methods section. Adjustments through linear regression and neural network models reconciled these values with their respective lithologies and geological units, enhancing their reliability^55,56^. After correction, the RMSE for porosity decreased from 3.65 to 1.68%, and the R2 increased from 0.422 to 0.832, based on a second-degree polynomial fit. Similar improvements were observed for other PPRs, such as P-wave velocity and bulk density (Fig. S2). These results are indicative of relatively high degree of reliability and consistency of the database, and ensure that the identified lithologies exhibit a stable behavior in relation to the studied variables. This level of consistency across lithologies establishes a strong foundation for predictive modeling and facilitates the accurate classification of lithofacies within this complex geological context^46^.

**Fig. 5:**
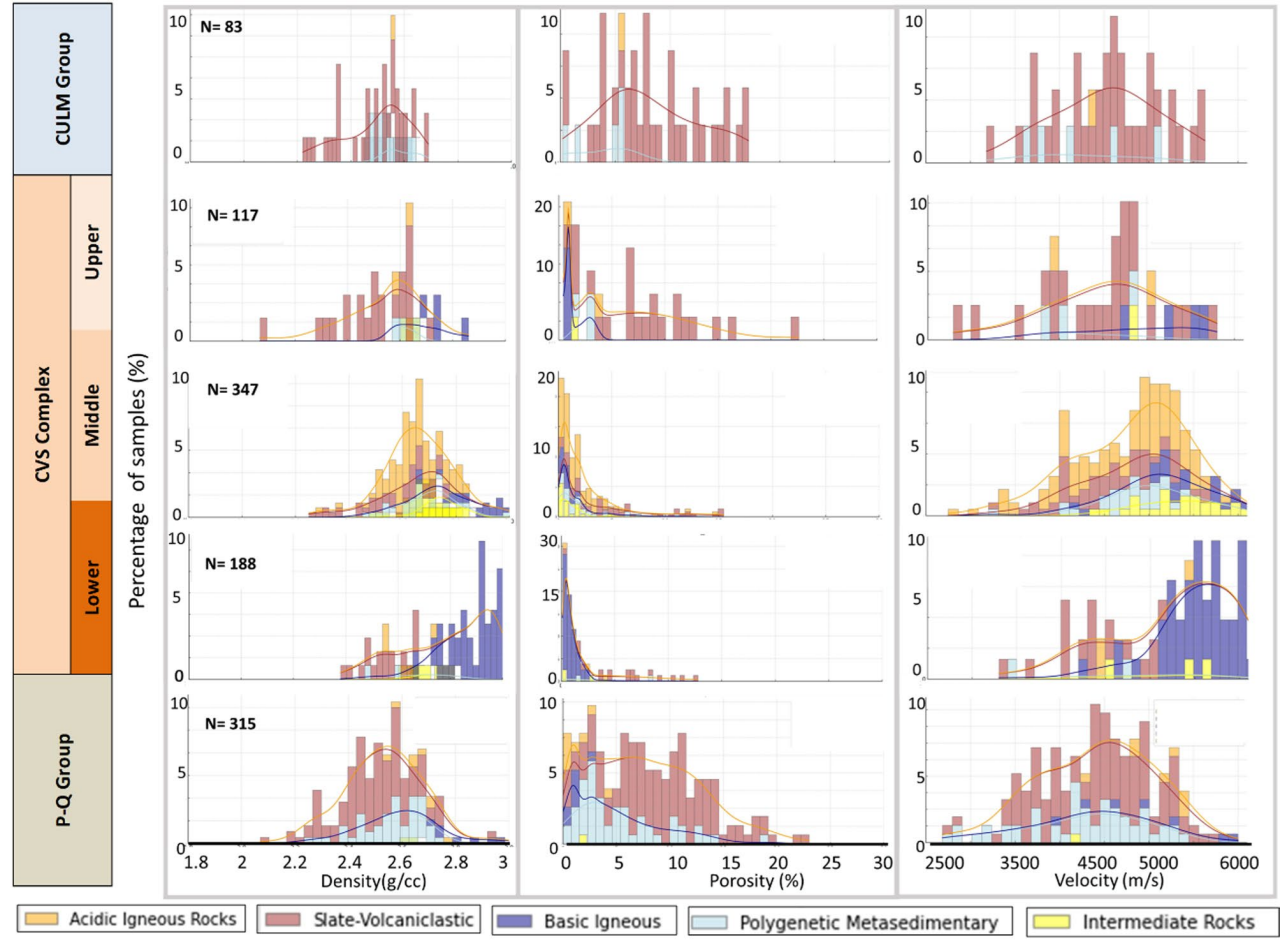
Distribution of PPR across the main geological units: bulk density (g/cm³), total porosity (%), and P-wave velocity (m/s). The color palette above the histograms corresponds to the five identified lithofacies. The figure highlights the overlap of PPR values ​​across geological units and lithofacies, demonstrating the variability and complexity of these properties within the studied context

Despite distinct lithological compositions, overlapping PPR ranges were observed across lithofacies. For example, Slate-Volcaniclastic lithofacies exhibited similar ranges of ρ and Vp to Basic and Intermediate Igneous lithofacies, regardeles their different composition as they consist of slates, basalts, and rhyolites, respectively. Such overlaps account for the limitations of single property approaches for PPR prediction and lithofacies classification in this geological setting. These findings reveal the need for a multi-variable analysis to improve accuracy and predictive capability^57–59^ (Fig. 5).

### Simple mathematical approach vs. ML

**Fig. 6:**
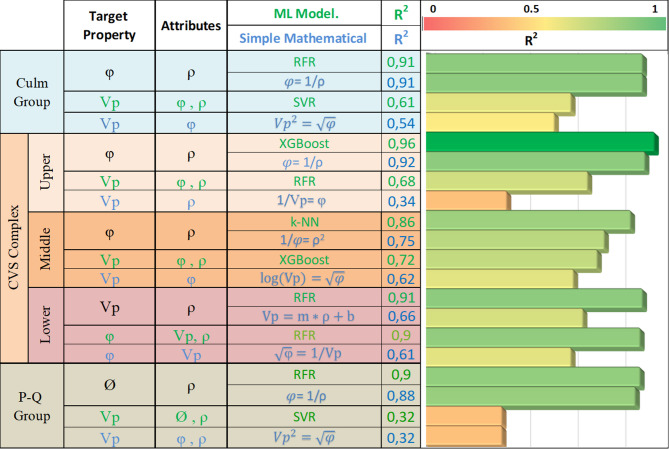
Comparison between the best simple mathematical and ML models for predicting PPR in each geological unit, with the R² values obtained by each model represented on a color scale. Where φ is total porosity (%); ρ is bulk density (g/cc) and Vp is P-wave velocity (m/s); RFR is Random Forest Regression; XGBoost is Extreme Gradient Boosting; K-NN is k-Nearest Neighbors and SVR is Support Vector Regression

A comparison of simple mathematical and ML models reveals key differences in their ability to identify relationships among ρ, φ, and Vp across geological units and lithofacies. Using simple models based on optimized functions, correlations exceeding R² = 0.50 were determined between variables for each geological unit (Fig. 6 and Table S2). These results align with previous studies and emphasize the importance of empirical relationships in understanding rock’s physical properties, particularly in sedimentary and volcanic lithologies^41,42,60^. For instance, in the P-Q Group, a robust correlation was observed between φ and the inverse of ρ (R² = 0.88). Within the CVS Complex, primarily composed of igneous rocks, the strongest correlation was between the inverses of φ and ρ^2^ (R² = 0.75).In the Culm Group, particularly in the Slate-Volcaniclastic lithofacies, the relationship between φ and ρ exhibited high stability (R² = 0.91). Additionally, a moderate correlation was noted between squared Vp and the square root of φ (R² = 0.54).

The ML models, however, showed superior predictive capabilities for PPR, especially in geological units characterized by higher ρ, faster Vp, and lower φ (Table S3). For the P-Q Group, RFR achieved remarkable accuracy in predicting φ from ρ (R² = 0.99), while Vp predictions were less effective due to the heterogeneity of the Slate-Volcaniclastic facies, with the best SVR model reaching R² = 0.32. In the CVS Complex, RFR excelled in the Lower CVS unit, accurately predicting Vp from ρ (R² = 0.91) and φ using both Vp and ρ (R² = 0.90). For the Middle CVS unit, XGBoost models performed best in predicting Vp (R² = 0.72), while KNN achieved the highest accuracy for φ (R² = 0.86). In the Upper CVS, φ predictions were highly accurate (R² = 0.96), though Vp predictions were less reliable (R² = 0.68) due to anisotropy and limited number of samples. In the Culm Group, RFR was very successful in predicting φ (R² = 0.91), but Vp predictions exhibited greater variability, with SVR achieving a maximum R² of 0.61, (Table S3).

Overall, the ML models demonstrated strong performance in predicting PPR across the geological units analyzed, especially in forecasting Vp, significantly outperforming traditional models without ML integration, This was supported by feature importance assessments derived from hierarchical models and permutation-based analyses, which consistently identified φ and ρ as the most influential predictors of Vp across the principal geological units (Fig. S3). Their combined influence enhanced model robustness and generalization by capturing nonlinear interactions and reducing prediction uncertainty (Fig. S3).

In terms of model interpretability, Shapley additive explanations (SHAP) bar and beeswarm plots (Fig. S6) were used to analyze the reverse relationship—estimating φ from physical proxies such as Vp and ρ. These analyses revealed that ρ is the dominant variable for predicting φ across all units, as evidenced by broader SHAP value ranges and higher mean contributions, while Vp showed more limited and stable effects. In low-porosity lithologies (< 5%), such as the Lower CVS, ρ also emerged as the primary predictor of Vp, indicating that in dense, compact rocks, elastic velocity is more strongly governed by ρ than by φ. These findings demonstrate that φ and ρ play dynamic, context-dependent roles as both, predictors and targets, and highlight the value of interpretable ML methods in supporting variable selection and enhancing trust in geologically coherent model outcomes.

Such capabilities have been demonstrated in previous studies where ML techniques like RFR and XGBosst effectively modeled complex geological scenarios and enhanced prediction accuracy in diverse mineral systems^1,61^. For instance the work of Thiéblemont et al.^53^. and Sun and Yang^56^ highlight the potential of combining supervised and unsupervised ML techniques for geophysical modeling. This aligns with the findings of this study, particularly in geological units characterized by significant heterogeneity. Similarly, Carranza et al.^62^ demonstrated the robustness of Random Forest models in handling sparse and noisy data, a critical aspect in geological datasets.In geological units dominated by Slate-Volcaniclastic lithofacies, the high variability of PPR, especially Vp, limited model accuracy, as seen in the P-Q, Upper CVS, and Culm units. (Fig. 6). This limitation is consistent with observations from previous works, where high variability in input features reduced the predictive performance of ML models^63^. Addressing these challenges through additional features such as mineralogical composition or advanced hybrid modeling approaches could further enhance the accuracy and applicability of these models^64,65^.

### ML rock type classification

The grouping of the 22 lithologies into 5 representative lithofacies of the IPB test site aims to simplify the geological model while highlighting key contrasts in PPR (Fig. S1a). Each lithofacies represents predominant lithologies and significant PPR differences (Fig. 5). Notably, total φ is much higher in the Slate-Volcaniclastic lithofacies, Vp increases significantly in the basic and intermediate igneous lithofacies. These PPR distinctions are crucial for using ML to effectively differentiate between the defined lithofacies^11,14^. The results from predicting lithofacies using unsupervised methods like K-Means and GMM were not promising, as illustrated by the low silhouette scores and diminishing clustering performance Figure. S4, inertia and silhouette analyses). Their limited performance is largely attributed to the significant overlap in physical property distributions, particularly among intermediate, acidic, and metasedimentary lithofacies, which prevented the models from forming robust clusters, as illustrated by the multiplot, principal component analysis (PCA), and t-distributed stochastic neighbor embedding (t-SNE) visualizations (Fig. S5).

As both methods achieved an average precision of around 20% in distinguishing the five main lithofacies categories. They were only able to classify the lithofacies of Basic Igneous and Slate-Volcaniclastic rocks with about 60% certainty, which are the most extreme lithofacies in terms of PPR values. In contrast, the classification certainty for intermediate lithofacies, such as Acid Igneous Rocks, Intermediate Rocks, and Polygenetic Rocks, was bellow 10%. The metasedimentary lithofacies, characterized by significant lithological and PPR heterogeneity, proved particularly challenging for the unsupervised models, which struggled to capture this complexity effectively^11,13^ (Fig. S5, S7).The results obtained from predicting the three primary lithofacies using supervised ML models, including RFC, ID3, CART, and KNN, revealed significantly improved performance compared to unsupervised methods. Evaluation metrics such as precision, recall, and F1 score highlighted CART as the most effective model for classifying the three main lithofacies (Slate-Volcaniclastic & Polygenetic Metasedimentary, Acidic & Intermediate Igneous Rocks, and Basic Igneous Rocks) (Table S5a, supplementary material).These findings align with the observed robustness of decision-tree-based models in similar applications, where CART has demonstrated superior performance in handling heterogeneous datasets^11^. The CART model achieved the highest F1 score 0.82, reflecting an optimal balance between precision and recall, with average values of 0.82 for both metrics. Conversely, KNN exhibited the poorest performance, with an average F1 score of 0.76 and precision and recall values of 0.78 and 0.74, respectively.

Confusion matrices for the applied ML models (Fig. S8, supplementary material) confirmed that RFC and CART yielded the best results for the three primary lithofacies. Slate-Volcaniclastic and Basic Igneous Rocks lithofacies consistently exhibited the highest classification precision, nearing 0.90 across models. This performance is consistent with their petrophysical behavior. Although internal lithological variations exist within each lithofacies these two groups represent the most pronounced and contrasting end-members within the sampled lithological variability^19,20^. Acidic & Intermediate Igneous Rocks lithofacies showed the lowest precision (0.72), attributed to their heterogeneity and greater overlap in PPR properties compared to other categories. These findings are consistent with the challenges of classifying heterogeneous lithofacies reported in other studies^12,14^. Based on these results, the CART model was selected for lithofacies classification, achieving an average precision of 82%.

**Fig. 7:**
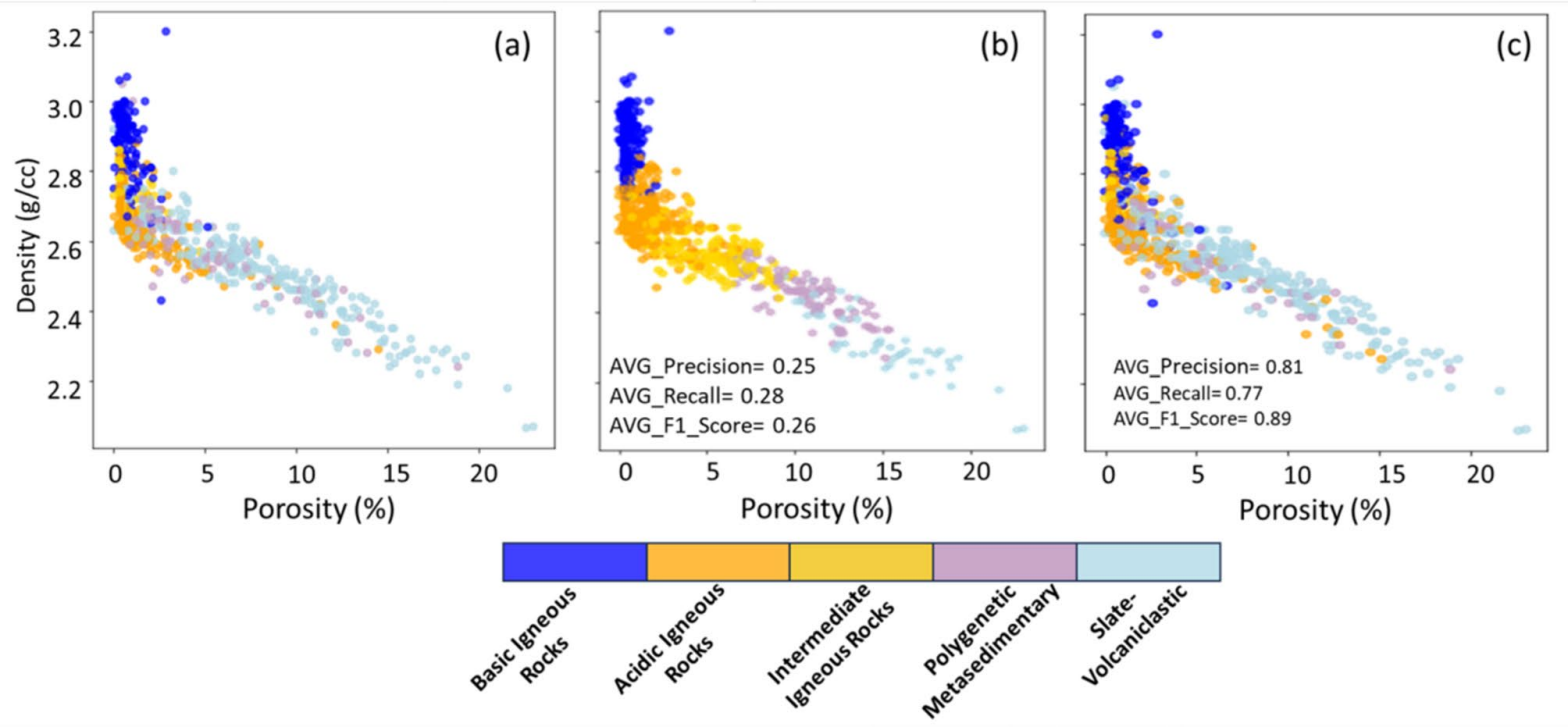
Comparison of lithological classification. **a** Real cross-plot with a color palette representing the distribution of the five defined lithofacies, **b** classification results from the best-performing unsupervised method, and **c** classification results from the best-performing supervised method.

After establishing classification models for three lithofacies, their performance in classifying the remaining two lithofacies: Polygenetic Metasedimentary and Intermediate Igneous Rocks, were assessed based on previously identified lithofacies: Slate-Volcaniclastic Metasedimentary and Acidic & Intermediate Igneous Rocks. The results indicated that the RFC models were the most effective, demonstrating strong metrics in distinguishing the Polygenetic Metasedimentary facies (F1 Score of 0.90, precision of 0.93, and recall of 0.87) and the Intermediate Igneous Rocks facies (F1 Score of 0.91, precision of 0.89, and recall of 0.87). In contrast, the KNN model performed the poorest in both categories, likely due to the dataset’s characteristics, which may require additional variables or attributes to improve the model’s robustness (Table S5b).These results are consistent with other applications of RFC in mining contexts, where its ability to handle high-dimensional datasets and rock type ranking has been highlighted^13,66^.The confusion matrices for this subclassification revealed consistent results, confirming the effectiveness of the strategy for reclassifying primary lithofacies. Additionally, it was noted that distinctive patterns in PPR were obscured when analyzing the entire dataset, which decreased the efficiency of ML algorithms in lithofacies discrimination (Fig. S8, S9). Figure 7 provides a summary of distinct cross-plots, comparing actual φ and ρ values, with a color palette representing the distribution of the five lithofacies. The figure reveals the superiority of supervised classification methods over unsupervised ones. Both numerically and visually, a strong correlation between the actual and predicted lithofacies can be observed, highlighting the enhanced accuracy and reliability of the supervised approaches^11,14^. Finally, regarding the QC results linked to the iterations with different random seeds 137 applied to the best RFC models, the comparison of: Precision, Recall, and F1 Score, metrics revealed variations below 5% (Fig. S10). These results stress the robustness of our methodological framework, illustrating that the integration of GridSearchCV, LOOCV, and stratified sampling ensures consistent and reliable performance, even when subjected to varying initial conditions.

### Prediction of PPR and classification lithofacies: the Riotinto case study

**Fig. 8:**
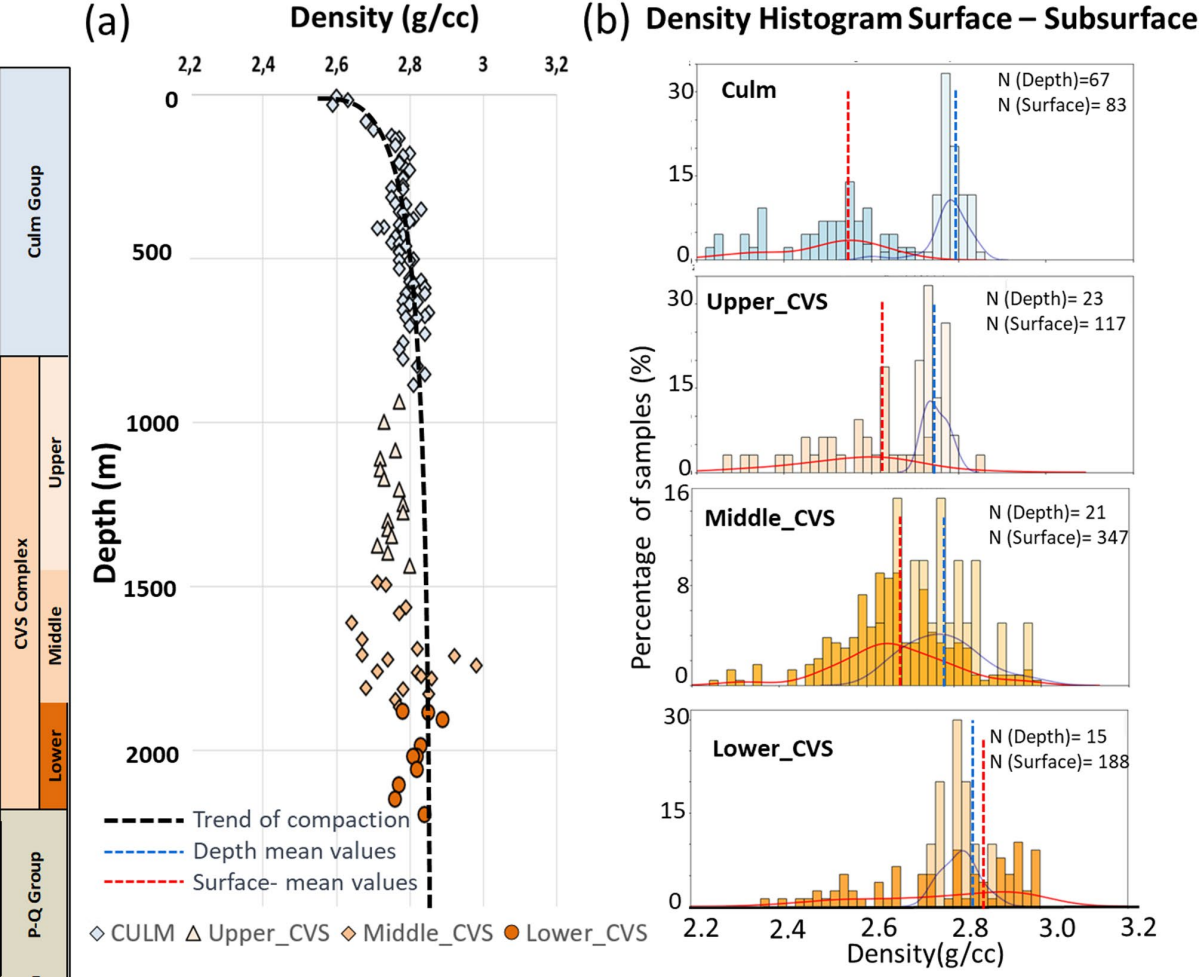
Variation of ρ with depth. **a** Values taken from boreholes. **b** Frequency histogram of ρ using surface samples and subsurface data for each geological unit

The analysis of subsurface-to-surface transfer functions highlighted distinct behaviors among geological units. Some units, such as the Culm Group and Upper CVS, demonstrated depth-related relationships with minimal dispersion in PPR values, enabling the derivation of mathematical functions to calculate property gradients (Fig. 8a). Conversely, units with low φ (below 5%) exhibited no significant depth-dependent relationships, as their subsurface-surface property histograms displayed consistent average measures (Fig. 8b). For the P-Q Group, which lacks borehole data, gradients were estimated using properties from the last 100 m of the Culm unit due to their similar lithologies and density distributions.

To address depth-related changes in PPR and mitigate biases associated with surface-only datasets, empirical transfer functions were derived for each geological unit. Second-degree polynomial functions (Table S6) provided the best fit for units such as the Culm Group and Upper CVS, characterized by relatively high φ and lower ρ and Vp values, indicative of progressive compaction or partial exhumation. These functions yielded robust metrics (average R² = 0.67), low RMSE and MAE, and trends consistent with the expected lithological evolution. For instance, density in the Culm Group increases with depth along a quadratic trend, eventually stabilizing as porosity is reduced by compaction.

Volcanic-dominated units like the Middle and Lower CVS showed negligible PPR-depth correlations, as suggested by the flat trends in Fig. 8b. This indicates that ML models trained on surface data remain valid for these units without requiring additional corrections. For the P-Q Group, the absence of borehole data was addressed by extrapolating gradients from the Culm unit, given their similar petrophysical signatures. Overall, these transfer functions proved essential for bridging surface and subsurface domains, enabling the refinement of ML models for more accurate borehole-scale predictions.

### Application of ML models to boreholes

**Fig. 9:**
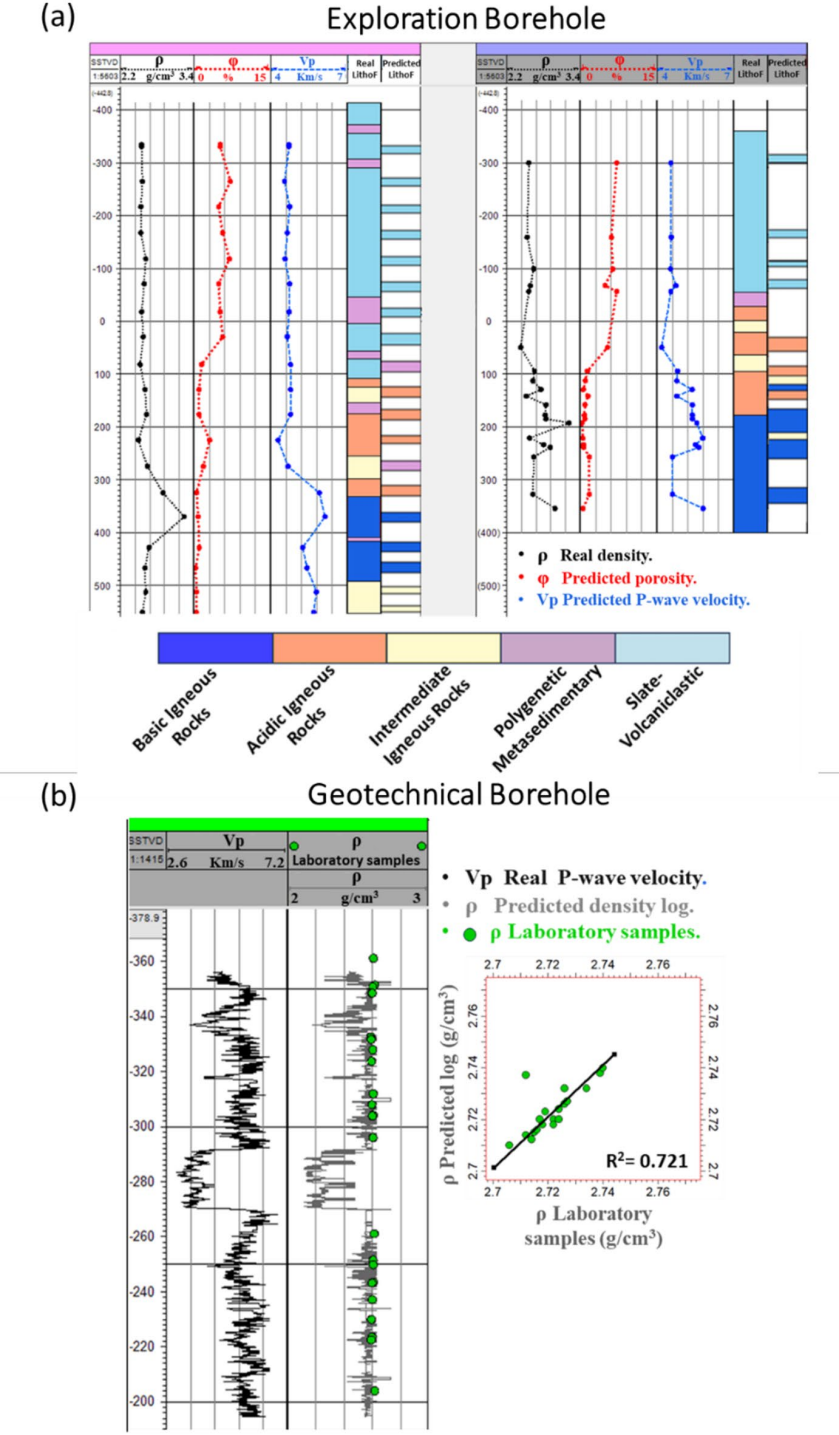
PPR and lithofacies predictions in Riotinto mine boreholes

The refined ML models were subsequently applied to both exploration and geotechnical boreholes at the Riotinto site. For the exploration boreholes, the models utilized real density values to predict Vp and φ. These predictions enabled the generation of complete datasets for each borehole, comprising density, porosity, and velocity values. These datasets were then used to classify three primary and two secondary lithofacies, achieving a correlation of 83% between the predicted and actual lithofacies described in the boreholes (Fig. 9a). The confusion matrices corresponding to these classifications are provided in Fig. S8 and S9, showing the distribution of correct and incorrect predictions across all lithofacies and supporting the reported accuracy. To provide a comprehensive overview, supplementary Figure S11 presents the results for all five remaining exploration boreholes, displaying measured and ML-predicted PPR alongside both core-described and predicted lithofacies, as well as the lithofacies classification accuracy for each borehole.Similarly, in the geotechnical borehole with a continuous Vp log, the ML models were employed to estimate density and porosity. These predicted values were validated against laboratory measured densities, achieving a precision of 72% (Fig. 9b).**a** PPR estimation and lithofacies classification in two exploration boreholes. Tracks show measured ρ (Track 1), ML-estimated φ and Vp (Tracks 2 and 3), real lithofacies from core analysis (Track 4), and ML-predicted lithofacies (Track 5).(b) Geotechnical borehole: Vp log (Track 1), laboratory-derived ρ (green points), ML-estimated ρ log (gray, Track 2), and an X-plot comparing laboratory and ML-predicted ρ values.

## Discussion

The multidisciplinary approach adopted in this study demonstrates that a dominant lithology can often be identified within each unit despite the geological units comprising multiple lithologies (Fig. S1). This facilitates the correlation of intervals with specific lithologies or lithofacies, simplifying the geological model. For instance, in the Riotinto case study, over 20 lithologies were grouped into five representative lithofacies, each dominated by a specific lithology consistent with the unit. This hierarchical classification not only streamlines the geological model but also reduces uncertainties by emphasizing key contrasts in PPR across lithofacies. This approach facilitates the integration of ML models, enhancing their predictive capabilities and enabling a more accurate characterization of subsurface geology in complex settings like the IPB^25,27,28^.

From a petrophysical perspective, the physical property ranges across geological units reveal significant overlaps, particularly for slate lithologies, which display the greatest variability^66^. This overlap challenges traditional modeling approaches, as properties like ρ and φ do not feature distinct ranges for each rock type. For instance, in the Riotinto area, the ρ of mafic igneous rocks closely resembles that of low-φ slates, and fractured basalts exhibit φ values similar to some slates. This complexity highlights the limitations of linear models and the need for ML based approaches, which can better capture the non-linear relationships, as demonstrated in previous studies^9,11,13^,15. Such methodologies allow for better handling of the heterogeneity inherent in geological units, providing a robust alternative to traditional linear models.

The overlap of properties highlights the importance of integrating multiple physical rock properties to achieve reliable subsurface models. ML approaches significantly enhanced prediction accuracy, particularly in lithologically heterogeneous units such as the Middle CVS. This improvement can be attributed to three key factors: (i) the detailed subdivision of geological units based on geological criteria, including temporal stratigraphic frameworks; (ii) the robustness of hierarchical ML models trained with physically and geologically meaningful features (e.g., ρ, φ, Vp) that showed strong statistical relevance with respect to the target variables; and rigorous quality control of data organized by lithology based data structuring, systematic outlier treatment, and cross-validation strategies. Together, these elements ensured that the models captured relationships that are not only statistically valid but also geologically coherent and interpretable, as also noted by Nguyen et al.^14^. Additionally, the staged prediction process further improved results, as detailed in the methodology Section. For instance, ML models achieved high predictive accuracy in the Lower and Middle CVS units, with R² values exceeding 80% for Vp predictions using ρ and total φ as input variables. However, performance was less robust in units dominated by slates (e.g., P-Q, Upper CVS, and Culm groups), likely due to the azimuthal anisotropy of Vp, a characteristic inherent to these lithologies^28,67^.

The analysis of simple mathematical relationships (Table S2 and Fig. 6), revealed that φ and ρ, as isotropic variables, generally exhibit a strong correlation. However, the relationships between φ and Vp or between ρ and Vp often deviate from linearity, particularly in scenarios influenced by factors specific to this study area, such as mineralogical composition, microstructure, and diagenetic processes^58^. This deviation emphasizes the value of ML models, which, as highlighted by previous studies^9,11,44,60^can effectively address the nonlinear interactions between these variables. Feature importance analyses derived from RFR models (Fig. S3) further support this interpretation, showing that φ and ρ are consistently among the most influential predictors of Vp across geological units, although their relative contributions vary according to lithology. For example, slates in the P-Q and Upper CVS groups, characterized by high φ values (> 9%), demonstrated minimal dispersion in φ–ρ correlations, largely due to the dominance of primary porosity contributions. However, Vp predictions based on these properties were less reliable, particularly in highly porous units, where the increased velocity heterogeneities within the rock could not be adequately captured by φ and ρ alone. ML models, on the other hand, effectively addressed these limitations by capturing the complex, nonlinear relationships between variables, even in cases with limited resolution in physical rock property measurements.

This improvement (Fig. 6) was particularly notable in the Lower and Middle CVS units, where ML models achieved R^2^ and estimations of Vp exceeding 80% using φ and ρ as predictor variables. These results reveal the strength of supervised ML models in capturing the non-linear relationships between PPRs, even in geologically complex environments^9,11,44^. These results are geologically consistent with the dominant lithologies and petrophysical characteristics of the Lower and Middle CVS units^19,20^. The Basic Igneous Rocks lithofacies, which largely corresponds to the Lower CVS, is composed mainly of massive basalt flows with low porosity, high density, and relatively uniform mineralogical and structural features. This lithofacies presents one of the most consistent petrophysical behaviors across the dataset, supporting robust and stable predictive relationships among ρ, φ, and Vp. In contrast, the Middle CVS is dominated by Acidic to Intermediate Igneous lithologies (e.g., rhyolites and dacites), which, while more variable in texture and composition, still maintain coherent geomechanical trends that enable effective modeling. Together, these two lithofacies represent contrasting endmembers in the lithological spectrum of the IPB, and their distinct petrophysical signatures enhance their separability in feature space.

In geologic units dominated by slates, such as the P-Q, Upper CVS, and Culm groups, the improvements remained less significant, indicating that the limitations may stem both from the predictor variables and, the azimuth dependence of the P-wave velocities in these rocks. The Lower CVS unit, mostly consisting of basaltic rocks, exhibits a more linear behavior of Vp about bulk ρ and total φ. This suggests that this unit exhibits less dispersion in geophysical properties compared to other units, which is an asset to be considered when interpreting geophysical data in the area. This reduced performance is consistent with the geological and petrophysical complexity of the P-Q Group^9^Upper CVS^19,20^and Culm^27,28^ units, all of which are dominated by slate-rich lithofacies. These lithologies exhibit elevated total porosity values, but with significant internal variability linked to microstructural anisotropy, foliation intensity, mineral alignment, and varying degrees of weathering or metamorphic overprint. Such factors affect the directional dependence of seismic velocities. These lithofacies also show overlapping PPR ranges with other units, further complicating their separability in feature space and limiting the performance of both regression and classification models.

The analysis of functional relationships among PPRs (Fig. 5) demonstrated significant overlap among the 22 identified lithologies, suggesting that the lithologies classification based on a single property can yield moderate accuracy. Grouping these lithologies into 3 to 5 lithofacies based on geological characteristics improved classification resolution to over 80% when using supervised ML models^11^. (Table S5). Hierarchical models like RFC and CART performed particularly well, achieving F1 scores above 0.80 and classification accuracy near 90%, especially for basic igneous and slate-volcaniclastic lithofacies.

In contrast, unsupervised methods such as K-Means and GMM (Fig. S4, S5,S6, S7) struggled to differentiate lithofacies with overlapping PPR values. Despite extensive parameter tuning, the clusters generated by both methods did not yield satisfactory results in terms of lithological classification. While they were able to reasonably distinguish extreme lithofacies classes, such as Basic Igneous Rocks and Slate-Volcaniclastic units, they failed to adequately resolve intermediate classes. This outcome highlights the inherent mineralogical complexity of the evaluated rock units, where the primary lithofacies lack distinct PPR ranges, further complicating their classification. Consequently, the clustering results were not incorporated into subsequent classification models. This reveals the limitations of unsupervised approaches in analyzing datasets characterized by significant overlap and variability, stressing the need for robust supervised methods to achieve reliable lithological classification Comparing ρ between surface and subsurface revealed a slight increase with depth in high φ lithologies, with minimal change (around 0.15 g/cm³) observed in the Middle and Lower CVS units (Fig. 8). While factors like meteoric exposure, confinement, and pressure may influence ρ, this study focused solely on depth variations. For the Culm, Upper CVS, and P-Q Group units, exponential functions best represent the property variations, whereas linear functions were more suitable for the other units^68^. Outcrop samples from the Lower CVS showed physical property values close to those at depth, contrasting with slates from the Culm, P-Q, and Upper CVS groups, where depth dependence was more pronounced.

The confusion matrices for the classification models (Figs. S3 and S4), along with the precision, recall, and F1-score metrics per lithofacies (Table S5), confirm the robustness and consistency of the classification results. In particular, the Slate-Volcaniclastic and Basic Igneous Rocks lithofacies achieved precision and recall values above 0.87 in most models, with F1-scores reaching up to 0.91. These high metrics indicate that the models reliably identify these classes when predicted based on physical rock properties (PPR), and also capture a large proportion of their actual occurrences. This performance is especially relevant in exploration contexts, as the ability to distinguish lithofacies with contrasting petrophysical signatures enables the identification of potentially favorable host units. If any of these lithofacies are associated with mineralized bodies, such as VMS, their contrast with surrounding units could further enhance the detection of geophysical anomalies. The consistency of precision and recall, combined with model stability, reinforces the applicability of this methodology to guide mineral exploration and reduce subsurface characterization uncertainty.

The application of ML-guided models to subsurface data, (including the data from the six exploration RT boreholes and a geotechnical borehole) reveal a robust performance, achieving 83% accuracy in classifying lithofacies compared to actual borehole descriptions (Fig. 9a). Additionally, the predicted ρ log showed over 72% in the geotechnical borehole (Fig. 9b) reliability when validated against laboratory measurements for PPR prediction, emphasizing the models’ robustness. These results reveal the capacity of ML approaches to integrate surface and subsurface data effectively, delivering detailed lithofacies classifications and reinforcing their potential for data-driven subsurface modeling in complex geological settings.

While more complex machine learning architectures, such as DL, may offer advantages in scenarios involving large-scale and unstructured data (e.g., images, time series, or natural language), their applicability in the current study is limited by both the nature and scale of the dataset. The data used here are structured, low-dimensional, and derived from quantitative physical measurements—conditions under which traditional, interpretable ML models have demonstrated a consistent and robust performance^12,13,16,17^. In this context, the methodology presented proves to be technically appropriate and highly effective, particularly in mining environments where data collection is often constrained by cost, accessibility, and geological complexity. The results show that careful data preprocessing, feature selection, and model tuning, traditional ML approaches can deliver reliable and generalizable predictions even in data-limited scenarios typical of mineral exploration.

In summary ML guided 1D models are a powerful tool for geological modeling, essential for resource exploration and reservoir characterization. Future work should focus on extending these models to 3D frameworks by integrating additional geophysical observables, such as seismic imaging and potential field data. This advancement would enhance spatial resolution and predictive accuracy, positioning these ML approaches as transformative tools for subsurface characterization and exploration strategies.

Given that the models were calibrated and validated using a dataset that integrates over 1,050 surface rock samples and records from six boreholes in the Riotinto area (northeastern IPB, Fig. 2), the results obtained can be considered representative of the petrophysical behavior of the main geological units within the study window. The lateral continuity of these units within the structural framework of the IPB, together with the lithofacies control, suggests that the identified patterns reflect robust variability ranges likely to persist in geologically equivalent areas.

For units such as the Upper CVS and the Culm Group, Fig. 5 indicates that the inclusion of at least 50 additional samples would be necessary to complete the statistical distribution expected from the normal behavior of each physical property. This limitation is also reflected in the lower prediction accuracy observed for some PPRs in these units. Aware of this, specific data interpolation techniques were applied to these formations, and a stratified sampling strategy was implemented during model training to preserve the proportional representation of the different lithologies across all subsets, as detailed in the Methodology section.

In this context, it is important to highlight the need to extend this methodological approach to other areas of the IPB, as well as to additional mining areas, through new data acquisition efforts (including rock physical properties, both discrete and continuous, downhole geophysical logs). The incorporation of logs would allow for higher-resolution characterization of petrophysical properties under true subsurface geological conditions, capturing vertical heterogeneities and enhancing the generalization capacity of the developed models. In this regard, the methodological framework presented here provides a solid and scalable foundation for future investigations in data-limited scenarios, facilitating its integration into multiparameter geological models and more advanced 3D predictive schemes.

## Conclusions

Mineral resource deposits are mostly located in highly complex geologic settings usually within hard rock environments, this is the case of the Riotinto within the IPB. Often in these highly heterogeneous scenarios, rocks feature a broad range of values for their PPR (ρ, φ, Vp in this case), which is mostly a result of the stress field (pressure and temperature) and their geodynamic history (metamorphism, diagenesis, water content, surface exposure, etc.). This makes it difficult to differentiate between lithologies based on the values of their physical properties. Nevertheless, ML models can find common patterns in this type of observables.

In this case study ML models such as RFR, XGBoost, KNN, and SVR can deduct Vp achieving high metrics (e.g. R^2^ of over 80% accuracy in Lower CVS and Middle CVS formations) where (φ and ρ) are used as effective predictor variables (with non-linear functionalities). The schemes were successful in grouping the over 20 different lithologies into just five dominant lithofacies only using three physical properties. Given the overlap/similarity of the rock’s physical property values among the different lithofacies (particularly in acidic, intermediate, and metasedimentary igneous facies) the supervised ML models were far superior to the unsupervised ones.

The development of surface-to-subsurface transfer functions has adjusted the dynamic range of previously established ML models, allowing them to more accurately reflect subsurface conditions. This methodology was validated using an independent dataset consisting of downhole logs (from exploration wells), enabling estimations of (ρ, Vp) for each geological unit. Furthermore, the methodology has the potential to upscale to 3D models by integrating geophysical observables such as gravity and seismic inversion data. This would enable volumetric predictions of rock properties, providing more accurate and detailed subsurface characterizations. In the context of the IPB, this could significantly enhance the understanding of mineral distribution and improve exploration strategies in complex geological settings.

Our findings highlight the importance of aligning methodological choices with both data characteristics and real-world project constraints. Rather than defaulting to complex architectures, this study shows that well established, carefully optimized ML techniques remain not only valid but highly effective in geoscientific applications, especially when working with structured, domain-specific data under limited sampling conditions. The approach presented here offers a practical and scalable solution that bridges current data limitations while laying the groundwork for future adoption of more advanced methods, including DL, as richer and more continuous datasets become available in the mining sector.

## Electronic supplementary material

Below is the link to the electronic supplementary material.

Supplementary Material 1

## Data Availability

The data used and analysed during the current study available from the corresponding author on reasonable request.

## Abbreviations

AEI: State agency for innovation
CART: Classification and regression trees
CVS: Volcanic-sedimentary complex
GMM: Gaussian mixture model
ID3: Decision tree classifier
IGME: Geological and mining institute of spain
IPB: Iberian pyrite belt
KNN k: Nearest neighbors
LOOCV: Leave-one-out cross-validation
MAE: Mean absolute Error
ML: Machine learning
MSCL: Multi-Sensor core logger
MSE: Mean squared error
PCC: Pearson correlation
PPR: Physical properties of rock
QC: Quality control
R2: Coefficient of determination
RFC: Random forest classification
RFR: Random forest regression
RT: Riotinto mine
SPZ: South portuguese zone
SVR: Support vector regression
SW: Southwest
TVD: True vertical depth
VMS: Volcanogenic massive sulfide
VP: P-wave velocity (seismic)
XGBoost: Extreme gradient boosting
φ: Porosity
ρ: Density
