# Supplementary material for: Machine learning in subsurface physical properties and lithofacies prediction in a mining context
Source: Sci Rep. 2025 Jul 21;15:26495. doi: 10.1038/s41598-025-11953-4 (PMC12279942; doi:10.1038/s41598-025-11953-4)
Supplement: Supplementary file 1 — Supplementary Material 1 [file 41598_2025_11953_MOESM1_ESM.pdf]

Supplementary material

Study	Year	Data Type	Target	ML Algorithms	Lithofacies Classification	Sparse Data Handling	Surface-Subsurface Integration
Sun & Yang	2023	Field + lab rock physics	Rock physics functions	Rational NN	✗	✗	✗
Xiang et al.	2020	Multi-layer GIS	Mineral potential (3D)	Random Forest	✗	✗	✗
Li et al.	2020	Surface geochemistry	Mineral potential	CNN, Transfer Learning	✗	✗	✗
Bui et al.	2020	Borehole logs	Slope failure	Hybrid ML	✗	✗	✗
Xu et al.	2019	Well logs + big petrophysical data	PPR pattern recognition, property prediction	Gradient Boosting, ANN, Ensemble	✓ (implied from logs)	✗	✗
Al-Mudhafar	2017	Well logs	Lithofacies + permeability	ANN, SVM	✓ (logs only)	✗	✗
Carranza et al.	2016	Borehole logs	Rock type	Random Forest	✓ (binary)	✗	✗
Díez-Montes et al.	2015	Surface petrophysics	3D geological model	Not ML-based	✗	✓	✗
This study	2025	Surface + borehole PPR	ϕ, ρ, Vp, lithofacies	RFR, SVR, XGBoost, CART	✓ (5 facies)	✓	✓

Table S1. Comparative overview of ML-based studies in mining geoscience. This table contrasts representative studies with the present work based on key methodological aspects: data type, target variable, ML algorithms used, lithofacies classification capabilities, sparse data handling, and whether surface–subsurface integration was performed. While previous studies typically focus on surface or borehole data in isolation, this study uniquely combines surface and subsurface petrophysical data, applies supervised ML models optimized for sparse datasets, and achieves robust lithofacies classification across five facies in the IPB.

Geological Unit		Order of Prediction	Target Variable	Attribute Variable	Optimizing Relationships	Metrics
CULM Group		1er	φ	ρ	$\varphi = 1/\rho$	MAE: 0.01<br>MSE: 0.02<br>R^2: 0.91
		2da	Vp	φ	$Vp^2 = \sqrt{\varphi}$	MAE: 127.87<br>MSE: 153453.87<br>R^2: 0.54
CVS Complex	Upper	1er	φ	ρ	$\varphi = 1/\rho$	MAE: 0.01<br>MSE: 0.02<br>R^2: 0.92
		2da	Vp	φ	$1/Vp = \varphi$	MAE: 383.85<br>MSE: 113262.27<br>R^2: 0.34
	Middle	1er	φ	ρ	$1/\varphi = \rho^2$	MAE: 0.013<br>MSE: 0.029<br>R^2: 0.75
		2da	Vp	φ	$\log(Vp) = \sqrt{\varphi}$	MAE: 273<br>MSE: 29543.56<br>R^2: 0.62
	Lower	1er	Vp	ρ	$Vp = m * \rho + b$	MAE: 278.83<br>MSE: 141343.74<br>R^2: 0.66
		2da	φ	Vp	$\sqrt{\varphi} = \frac{1}{Vp}$	MAE: 0.17<br>MSE: 0.346<br>R^2: 0.61
PQ Group		1er	φ	ρ	$\varphi = 1/\rho$	MAE: 0.02<br>MSE: 0.06<br>R^2: 0.88
		2da	Vp	φ	$Vp^2 = \sqrt{\varphi}$	MAE: 463.86<br>MSE: 204697.87<br>R^2: 0.32

*MAE: Mean Absolute Error *MSE: Mean Squared Error. *R^2: Coefficient of Determination.

Table S2. Summary table of simple mathematical relationships between analyzed PPR for the estimation of the target variable from the attribute variable.

		Objective Variable	Attribute Variable	Best ML Model	Hyperparameters	Optimal Values	Metricas
Culm Group		φ	ρ	RFR	Minimum samples split	2	MAE: 0.02
					Minimum samples leaf	1	MSE: 0..12
					Maximum tree depth	15	R^2: 0.91
		Vp	φ, ρ	SVR	Kernel	Polynomial	MAE: 203.72
					Regularization	20	MSE: 37302.53
					Gamma	0.0125	R^2: 0.60
CVS Complex	Upper	φ	ρ	XGBoost	n_estimators	25	
					Learning rate	0.1	MAE: 0.052
					Maximum child weight	10	MSE: 0.37
					Min_samples_leaf	1	R^2: 0.96
					Maximum tree depth	2	
		Vp	φ, ρ	RFR	Minimum samples split	3	MAE: 459.07
					Minimum samples leaf	2	MSE: 113842.49
					Maximum tree depth	15	R^2: 0.68
	Middle	φ	ρ	KNN	N_neighbors	5	
					weights	uniform	MAE: 0.09
					algorithm	auto	MSE: 0.18
					leaf_size	20	R^2: 0.86
					distance type	Euclidiana	
		Vp	φ, ρ	XGBoost	n_estimators	25	
					Learning rate	0.1	MAE: 173.02
					Maximum child weight	5	MSE: 25025.34
					Min_samples_leaf	4	R^2: 0.72
					Maximum tree depth	2	
	Lower	Vp	ρ	RFR	Minimum samples split	2	MAE: 96.45
					Minimum samples leaf	1	MSE:41987.22
					Maximum tree depth	20	R^2: 0.91
		φ	Vp, ρ	RFR	Minimum samples split	2	MAE: 0.12
					Minimum samples leaf	2	MSE:0.25
					Maximum tree depth	20	R^2: 0.90
PQ Group		φ	ρ	RFR	Minimum samples split	3	MAE: 0.01
					Minimum samples leaf	1	MSE: 0.09
					Maximum tree depth	15	R^2: 0.86
		Vp	φ, ρ	SVR	Kernel	Linear	MAE: 329.53
					Regularization	5	MSE: 117302.53
					Gamma	0.0125	R^2: 0.41

*MAE: Mean Absolute Error *MSE: Mean Squared Error. *R^2: Coefficient of Determination.

Table S3. Range of Hyperparameters used in the PPR Prediction for each of the best ML models.

(a) **Unsupervised Methods**

Method	Hyperparameter	Range of Values
GMM	n_components	2–10
	covariance_type	'full', 'tied', 'diag', 'spherical'
	random_state	42
	init_params	'kmeans', 'random'
K-Means	n_clusters	2–7
	init	'k-means++'
	random_state	1
	n_init	10–50

(b) **Supervised Methods**

Method	Hyperparameter	Range of Values
RFC	min_samples_split	2–30
	min_samples_leaf	0.5–15
	max_depth	5–30
	n_estimators	100–500
	max_features	'sqrt', 'log2', None
	criterion	'gini', 'entropy'
	class_weight	'balanced', None
ID3	criterion	'gini', 'entropy'
	max_depth	None, 5–30
	min_samples_split	2–10
	min_samples_leaf	1–10
CART	criterion	'gini', 'entropy'
	max_depth	None, 5–30
	min_samples_split	2–10
	min_samples_leaf	1–4
	max_features	'sqrt', 'log2', None
	ccp_alpha	Cost complexity pruning
KNN	n_neighbors	3–7
	weights	'uniform', 'distance'
	metric	'euclidean', 'manhattan', 'minkowski'
	algorithm	'auto', 'ball_tree', 'kd_tree', 'brute'
	leaf_size	10–50

Table S4. Parameters used for lithology classification models. (a) unsupervised methods (K-Means, GMM). (b) supervised (RFC, CART, KNN). The table provides hyperparameter configurations, including maximum depth, minimum sample split, number of clusters, and initialization strategies.

(a)

Main 3 lithofacies	RFR			ID3			CART			KNN		
	Precision	Recall	F1-score	Precision	Recall	F1-score	Precision	Recall	F1-score	Precision	Recall	F1-score
Slate-Volcaniclastic & Polygenetic Metasedimentary	0,84	0,83	0,83	0,86	0,87	0,86	0,85	0,88	0,86	0,78	0,84	0,81
Acidic & Intermediate Igneous Rocks	0,72	0,76	0,74	0,72	0,73	0,72	0,75	0,74	0,74	0,70	0,59	0,64
Basic Igneous Rocks	0,92	0,74	0,82	0,79	0,77	0,78	0,85	0,83	0,84	0,85	0,79	0,82
Average	0,83	0,78	0,80	0,79	0,79	0,79	0,82	0,82	0,82	0,78	0,74	0,76

(b)

Secondary lithofacies (division of Main	RFR			ID3			CART			KNN		
	Precision	Recall	F1-score	Precision	Recall	F1-score	Precision	Recall	F1-score	Precision	Recall	F1-score
Slate-Volcaniclastic	0,95	0,91	0,93	0,89	0,85	0,87	0,84	0,80	0,82	0,85	0,81	0,83
Polygenetic Metasedimentary	0,93	0,87	0,90	0,87	0,82	0,84	0,82	0,77	0,79	0,84	0,79	0,81
Acidic Igneous Rocks	0,93	0,91	0,92	0,89	0,87	0,88	0,84	0,82	0,83	0,83	0,81	0,82
Intermediate Igneous Rocks	0,91	0,87	0,89	0,85	0,84	0,84	0,79	0,78	0,78	0,78	0,77	0,77
Average	0,93	0,89	0,91	0,88	0,85	0,86	0,82	0,79	0,81	0,83	0,80	0,81

Table S5. Performance metrics of each ML model used in the prediction of the 5 lithofacies. The upper table shows the metrics for the 3 main lithofacies, while the lower table details the subdivision and performance for the remaining two lithofacies.

Unit	Variable	Model	Transfer Function	R^2	RMSE	MAE
CULM	ρ	Polinómico grado 2	$\rho = -0.00001 * z^2 + 0.00043 * z + 2.669$	0.81	±0.06 g/cm^3	±0.05 g/cm^3
CULM	φ	Polinómico grado 2	$\phi = 0.00001 * z^2 + -0.01500 * z + 7.294$	0.76	±1.0 %	±0.8 %
CULM	Vp	Polinómico grado 2	$Vp = -0.00079 * z^2 + 1.02941 * z + 4600.811$	0.62	±280 m/s	±220 m/s
Upper_CSV	ρ	Polinómico grado 2	$\rho = 0.00002 * z^2 + -0.00065 * z + 3.115$	0.67	±0.08 g/cm^3	±0.07 g/cm^3
Upper_CSV	φ	Polinómico grado 2	$\phi = -0.00001 * z^2 + 0.02239 * z + -8.130$	0.59	±1.2 %	±1.0 %
Upper_CSV	Vp	Polinómico grado 2	$Vp = 0.00072 * z^2 + -1.66700 * z + 6044.803$	0.56	±350 m/s	±280 m/s

Table S6: Polynomial transfer functions used to adjust PPR values by depth in selected geological units. Second-degree polynomial models were fitted for the Culm Group and Upper CVS units, capturing depth-related trends in ρ, φ, and Vp. The table presents the model type, fitted function, R^2, and associated error metrics (RMSE and MAE).

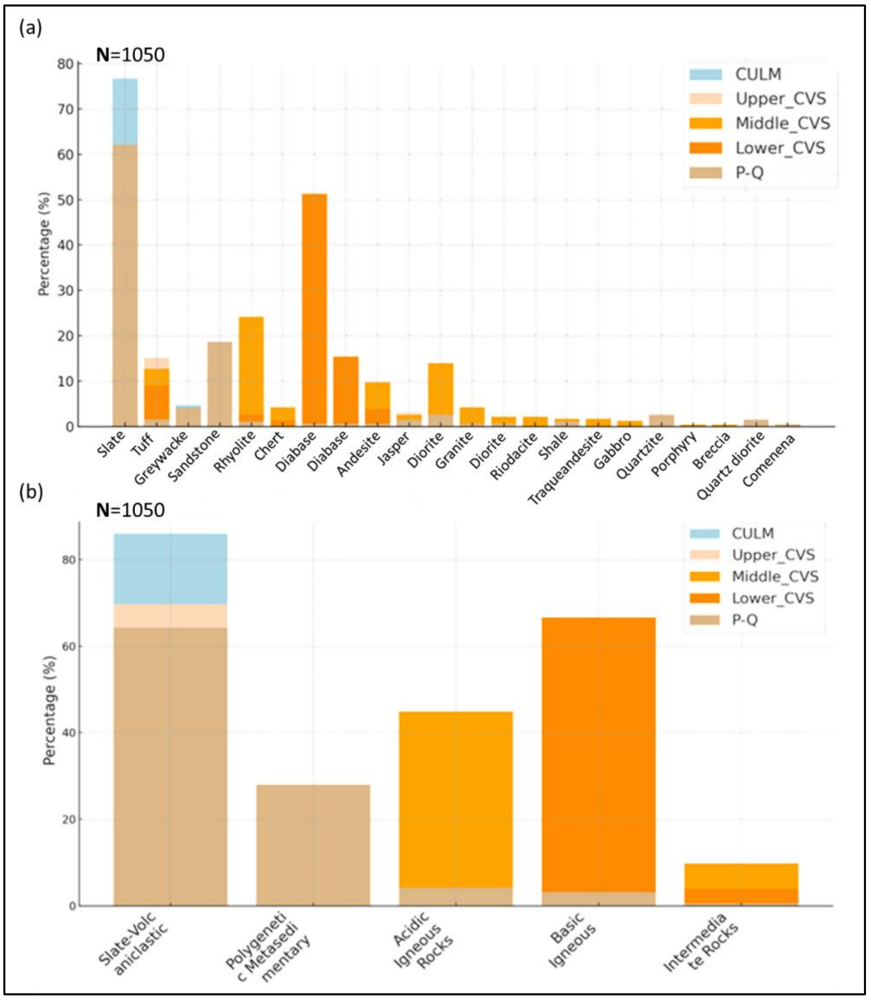

Figure S1. Lithology and Lithofacies distribution across geological units. (a) Frequency histogram of the different lithologies of the studied samples, with colors indicating the corresponding geological units. (b) Frequency histogram grouped by lithofacies, with colors indicating the main geological units.

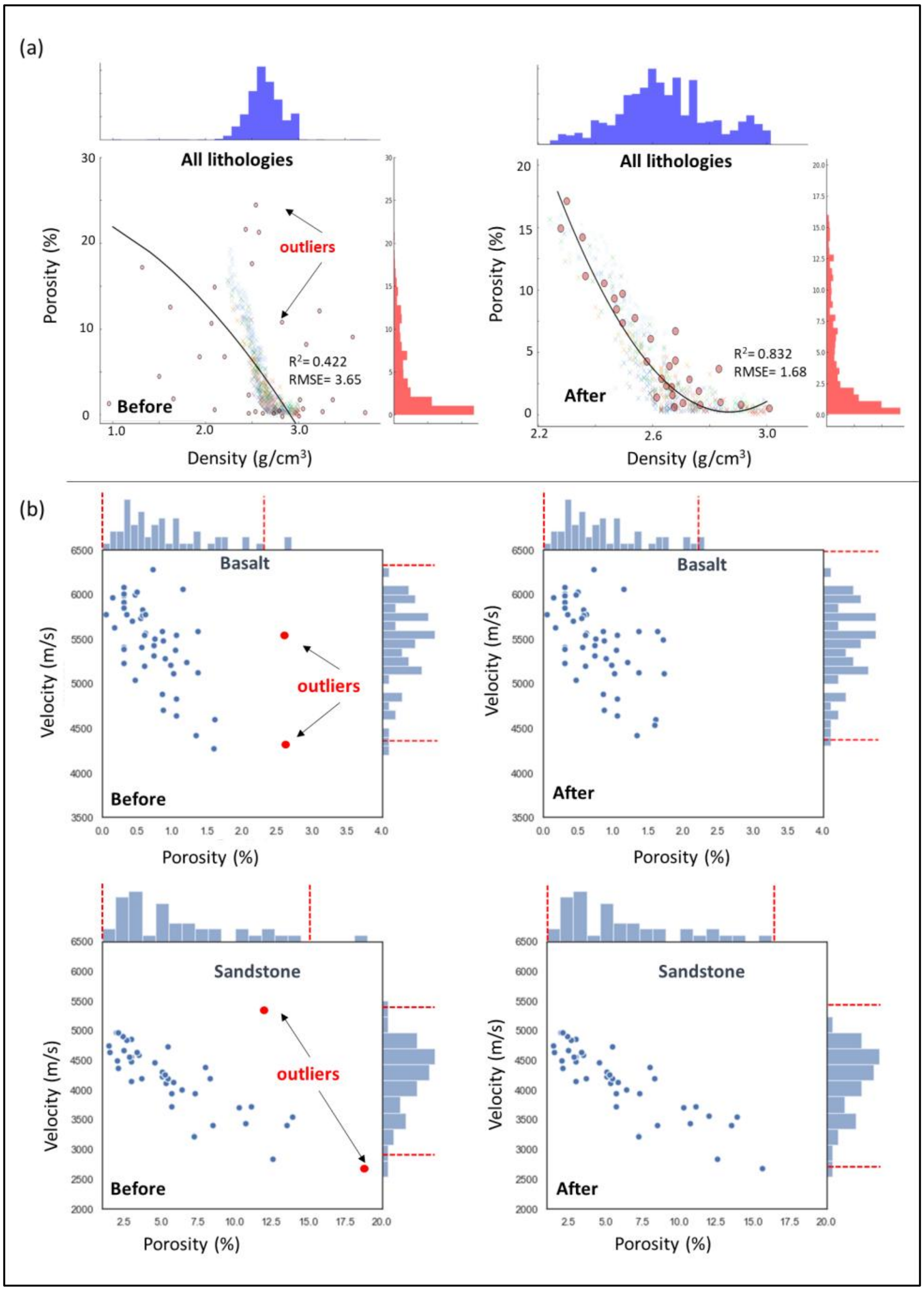

Figure S2. Identification and correction of outliers. (a) Example of total outlier detection across all lithologies, showing data dispersion before and after correction using neural networks. (b) Illustration of the correction process for two specific lithologies, basalt (top) and sandstone (bottom). In both cases, corrected values align more closely with the global and lithology-specific trends, enhancing consistency and reliability**.**

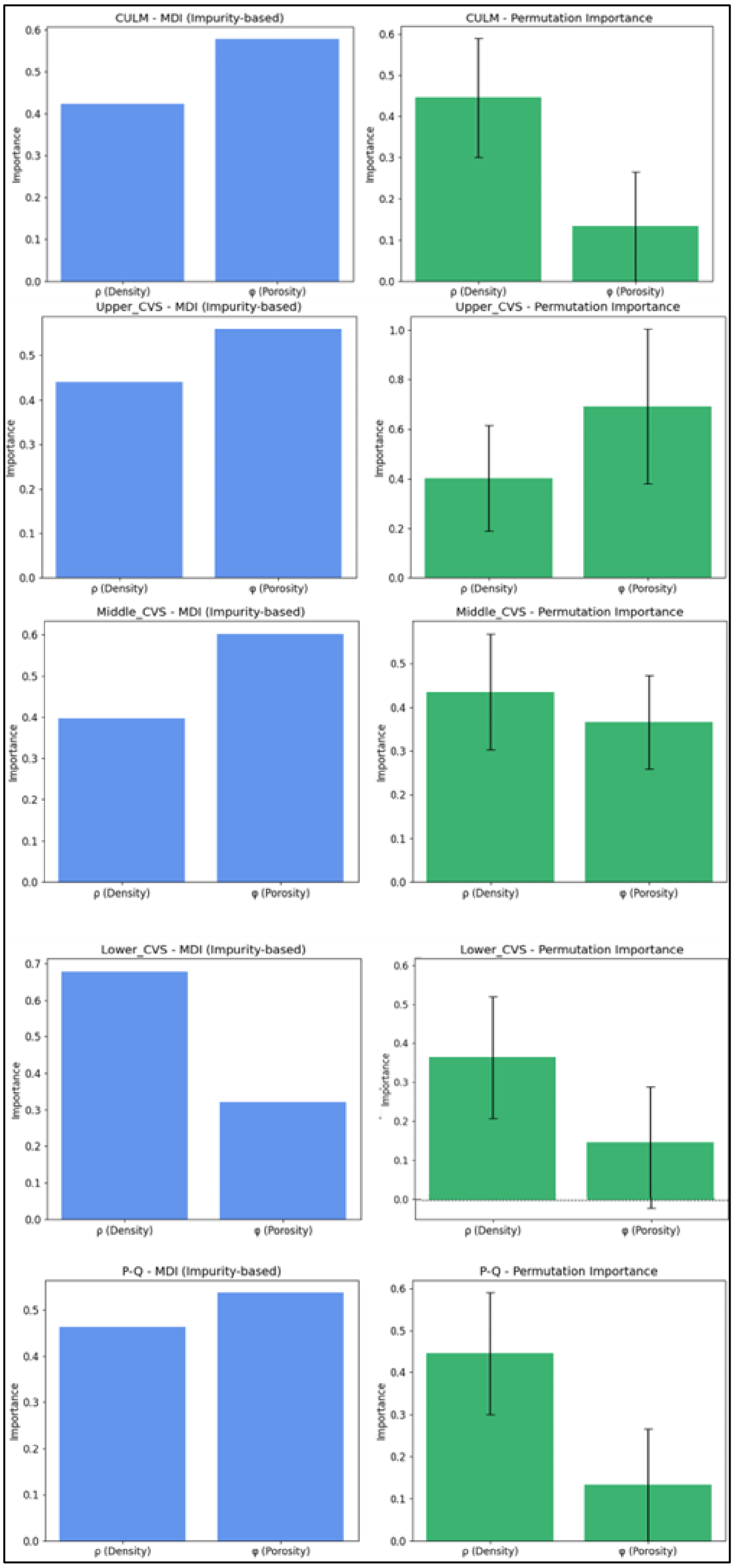

Figure S3. Feature importance for Vp prediction across geological units. Bar plots showing the relative importance of ρ and φ in predicting Vp, derived from RFR. Left column: Mean Decrease in Impurity, (MDI); right column: permutation-based importance with error bars. Results are shown for each principal geological unit: Culm, Upper CVS, Middle CVS, Lower CVS, and P-Q Group. The analysis confirms that both φ and ρ are influential predictors of Vp, with their relative contributions varying according to the lithological characteristics of each unit.

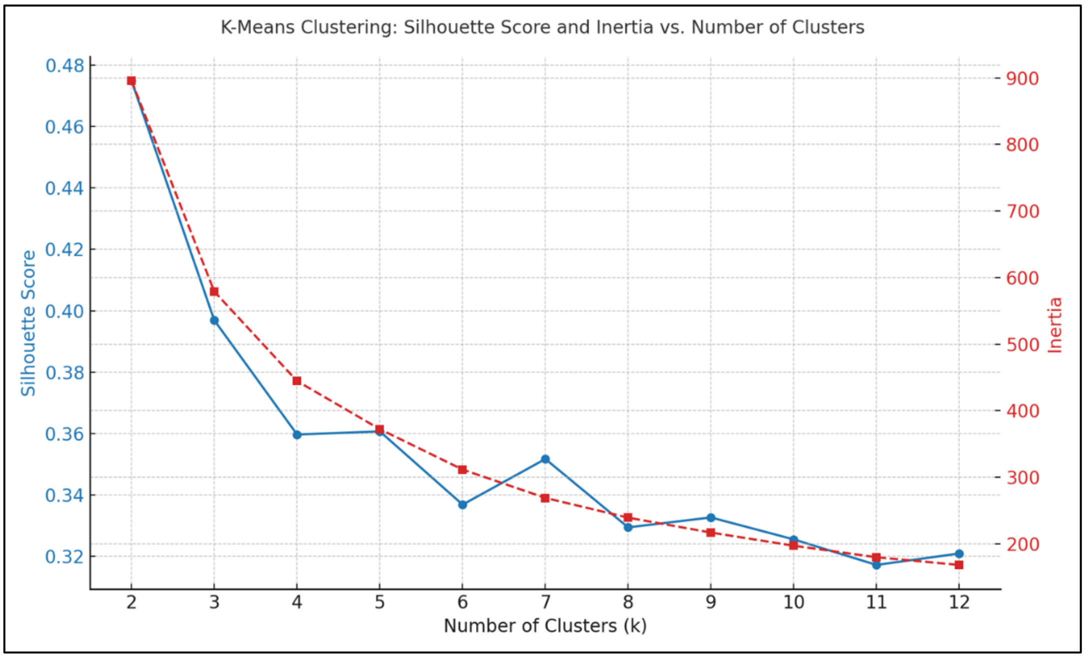

Figure S4: . Combined analysis of silhouette coefficient and inertia for K-Means clustering using PPR (ρ, φ, Vp). While inertia consistently decreases with increasing number of clusters, silhouette scores peak at K=3 (0.27), then drop progressively, indicating diminishing cluster separation. The results highlight the limited clustering structure in the data due to strong overlap in PPR distributions across lithofacies.

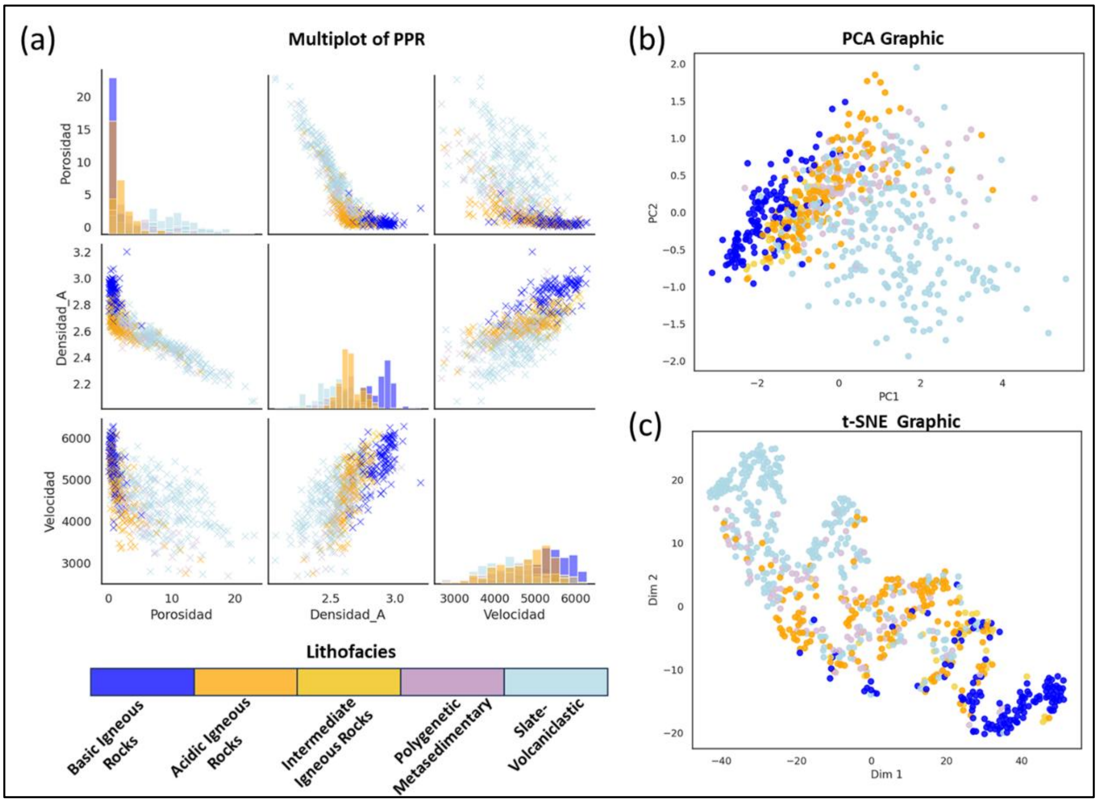

Figure S5. Visualization of PPR overlap across principal lithofacies**.** (a) Multiplot matrix showing pairwise relationships among φ, ρ, and Vp across the five main

lithofacies. (b) Principal Component Analysis (PCA) projection. (c) t-distributed Stochastic Neighbor Embedding (t-SNE) projection. All visualizations highlight the substantial overlap in physical property space among intermediate, acidic, and metasedimentary lithofacies, which accounts for the limited performance of unsupervised classification models such as K-Means and GMM.

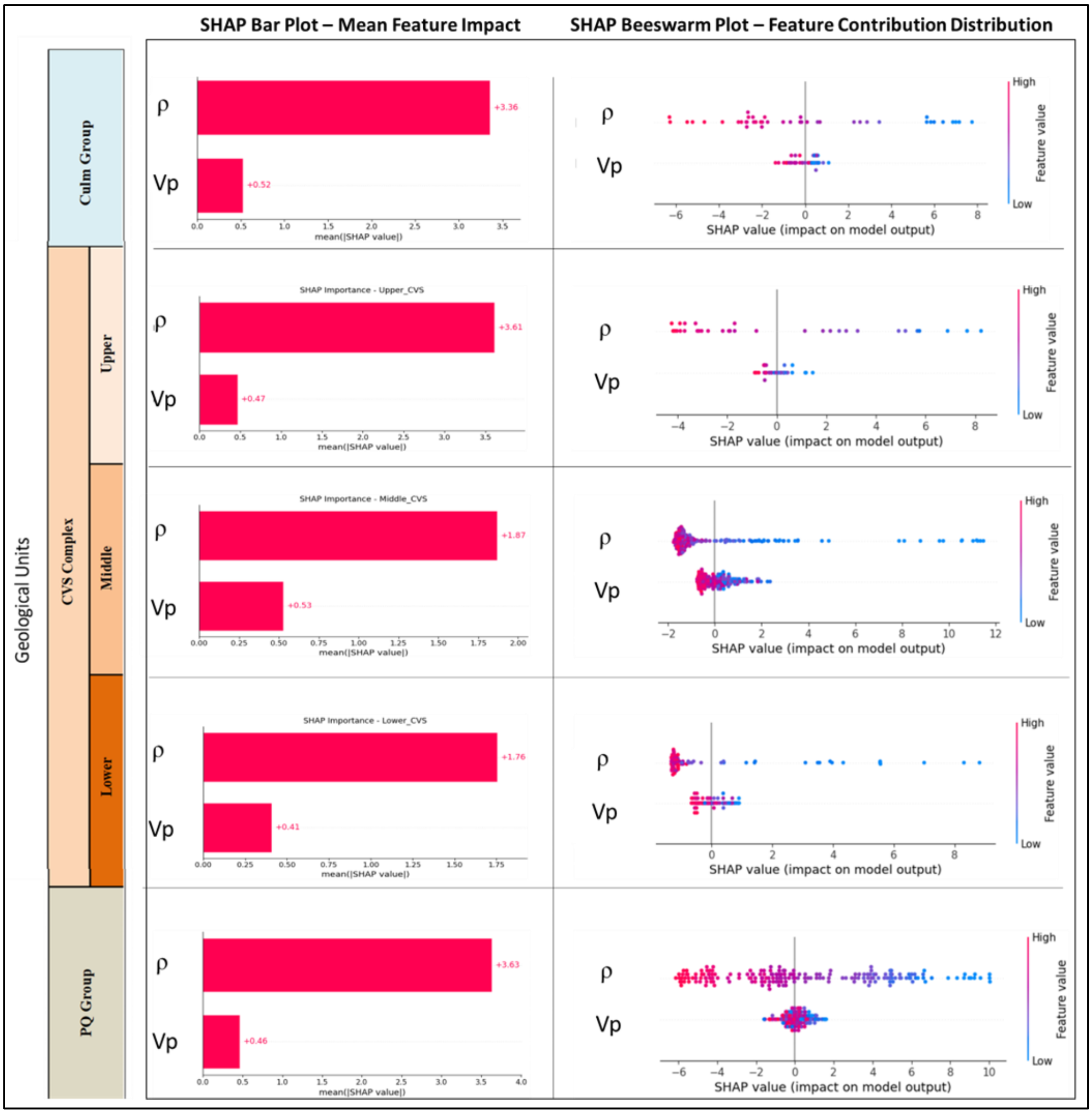

Figure S6. SHAP analysis for model interpretability by geological unit. Left: Mean SHAP value bar plots showing the relative importance of ρ and Vp in predicting φ across the main geological units. Right: Beeswarm plots illustrating the distribution of SHAP values per feature, with color indicating the feature value. These plots confirm that ρ is generally the most influential predictor of φ, with its impact especially prominent in dense units such as Lower CVS. Conversely, Vp shows a more limited and stable contribution across units.

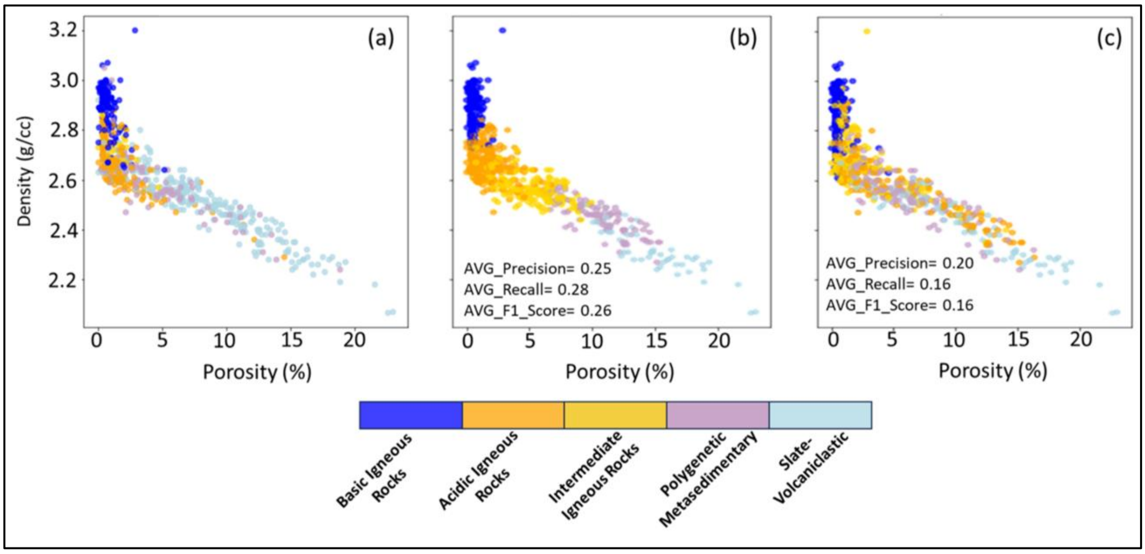

Figure S7. Unsupervised classification methods. Cross-plots of density versus porosity for all geological units, with a color-coded palette representing the five defined lithofacies. (a) Actual lithofacies classification based on observed data; (b) predicted lithofacies classification using K-Means clustering; (c) predicted lithofacies classification using the Gaussian Mixture Model (GMM).

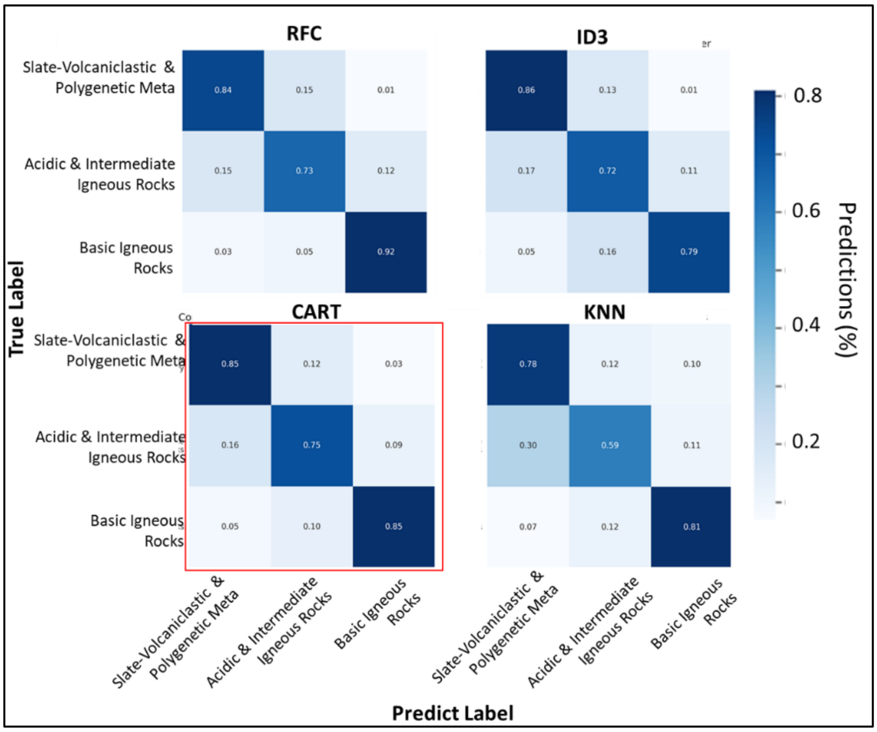

Figure. S8. Confusion matrices depicts the performance of ML classification model in estimating the three main lithofacies. This representation especially highlights the CART model (red box) for its outstanding performance.

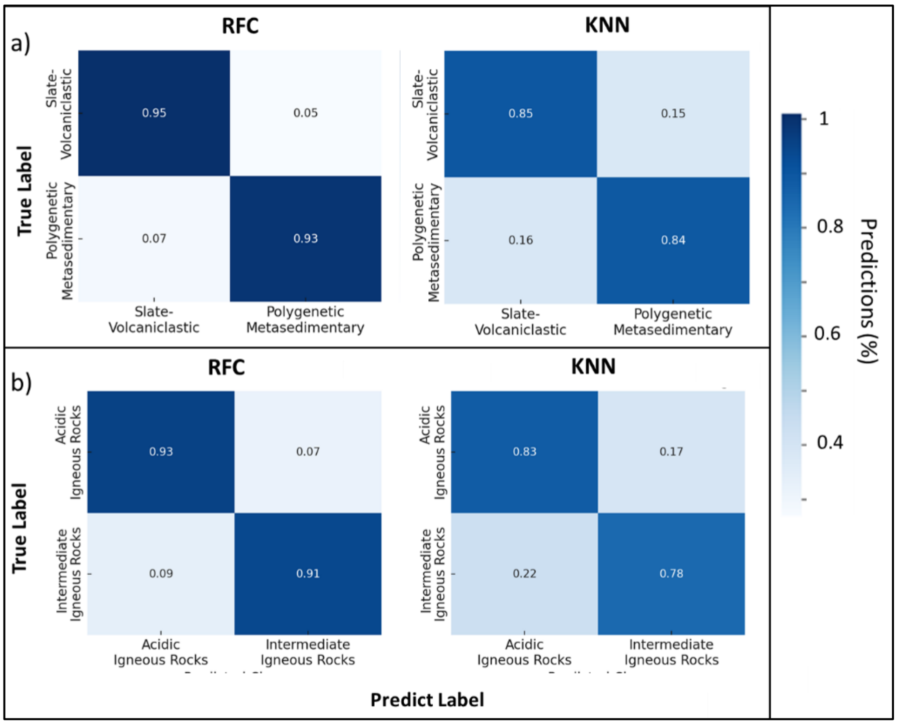

Figure. S9. Confusion matrices for the ML models with the highest performance (RFC) and lowest performance (KNN) in predicting the secondary lithofacies 'polygenetic metasedimentary' and 'intermediate igneous.

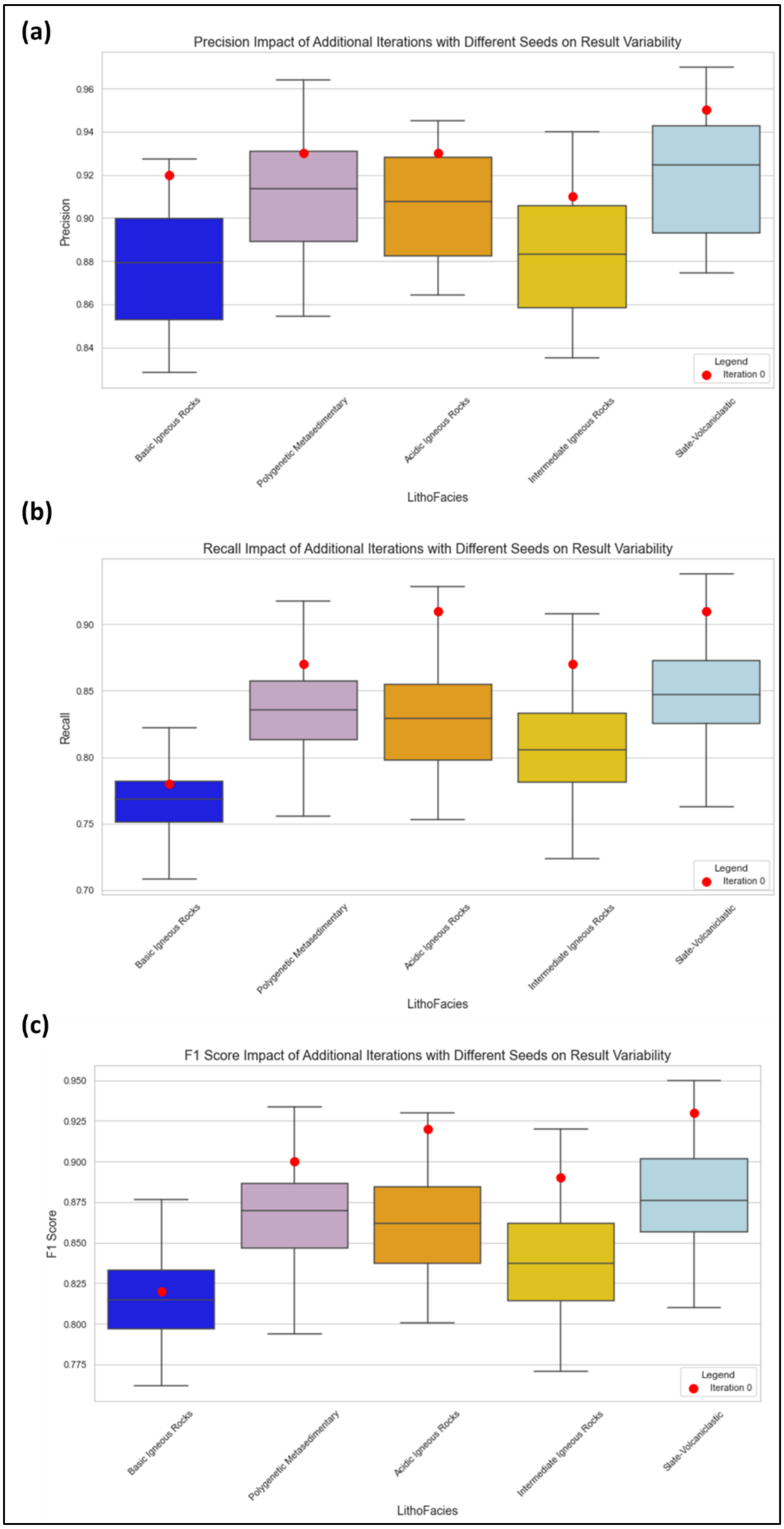

Figure S10. Comparison of 137 iterations using different random seeds in the RFC method versus the results of iteration 0 (red point), considering hyperparameter optimization with GridSearchCV and LOOCV. The figure shows a) Precision, b) Recall, and c) F1 Score across iterations, confirming the robustness of the methodology due to data diversity, exhaustive evaluation, and optimal parameter tuning.

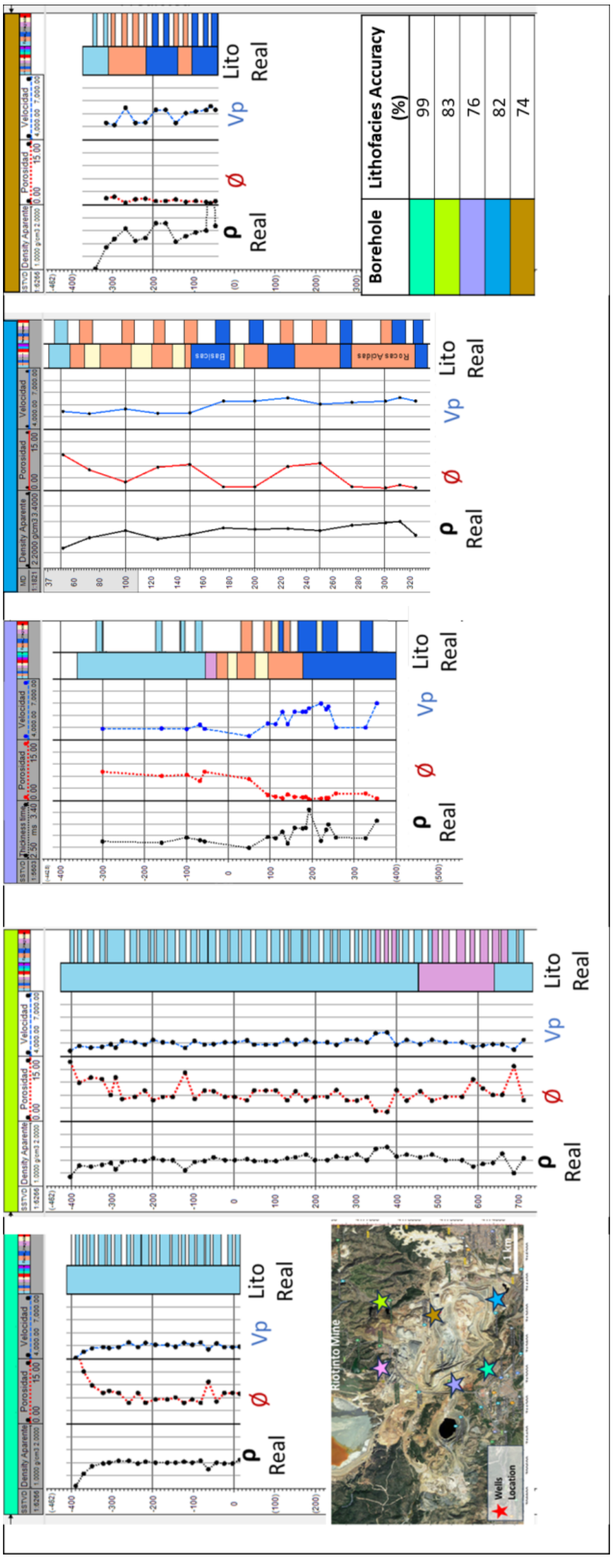

Figure S11. PPR estimation and lithofacies classification in all six exploration boreholes from the Riotinto study area. Each panel displays five tracks: measured density ρ (Track 1), ML-predicted porosity φ (Track 2) and P-wave velocity Vp (Track 3), lithofacies interpreted from core descriptions (Track 4), and ML-predicted lithofacies (Track 5). Color-coded bars indicate lithofacies categories. Borehole identifiers are anonymized using color codes, and their geographic locations are shown on the satellite map (bottom left). The lithofacies classification accuracy per borehole ranges from 76% to 88%, supporting the robustness and generalization capability of the applied ML models.
